# Supplementary material for: A connexin/ifi30 pathway bridges HSCs with their niche to dampen oxidative stress
Source: Nat Commun. 2021 Jul 23;12:4484. doi: 10.1038/s41467-021-24831-0 (PMC8302694; doi:10.1038/s41467-021-24831-0)
Supplement: Supplementary file 1 — Supplementary Information [file 41467_2021_24831_MOESM1_ESM.pdf]

Supplementary tables

Table S1: primers used for genotyping transgenic and mutant embryos, cloning of *ifi30* full-length mRNA and to produce ISH probes.

Gene	Forward	Reverse
UAS	AAAACTAGTCGTGTGGAGGAGCTCAAAG	AAAGCGGCCGCGGGATCACGCGGCCATCA
Gal4	GCTACTGTCTTCTATCGAACAAGC	TGCTGTCTCAATGTTAGAGGC
ifi30^{sa19758} (mutant)	AGTGACTCCTGTACCAAAAC	TACCTGTGCGTTTCCATATG
ifi30 mRNA-injection	AAAGGATCCCATCATGTTCGGCTTTAACC	AAACTCGAGCCCTGATTAGTTCATGCAGT
ifi30 probe	TTAACCTGTGCGTTGTGCTC	CAGCTGGTTTTTGACCCTTG
cx41.8 $^{t1\ t1}$ (mutant)	AGGTTAATTGGCCAAATTAGGC	AGATCAGAGAAGGTGTAGAC

Table S2: morpholino-oligonucleotides and primers used to test morpholino knock-down efficiency by reverse transcription polymerase chain reaction (RT-PCR).

Standard control-MO	CCTCTTACCTCAGTTACAATTTATA	
ifi30-MO	CGTGCAGTAGTGTAAATATACCTGT	
**Gene**	**Forward**	**Reverse**
ifi30	CATCATGTTCGGCTTTAACC	CCAGACATTCATCTTCTCCG

Table S3: primers used for qPCR

**Gene**	**Forward**	**Reverse**
ifi30 qPCR	GGCTTGGATGCTGTTATGGT	CACGGCACATATTGATGAGG
cx41.8$^{t1/t1}$ qPCR	AGATCAGAGAAGGTGTAGAC	AGGTTAATTGGGCAAATTAGG
eif1a qPCR	GAGAAGTTCGAGAAGGAAGC	CGTAGTATTTGCTGGTCTCG

Supplementary figure 1

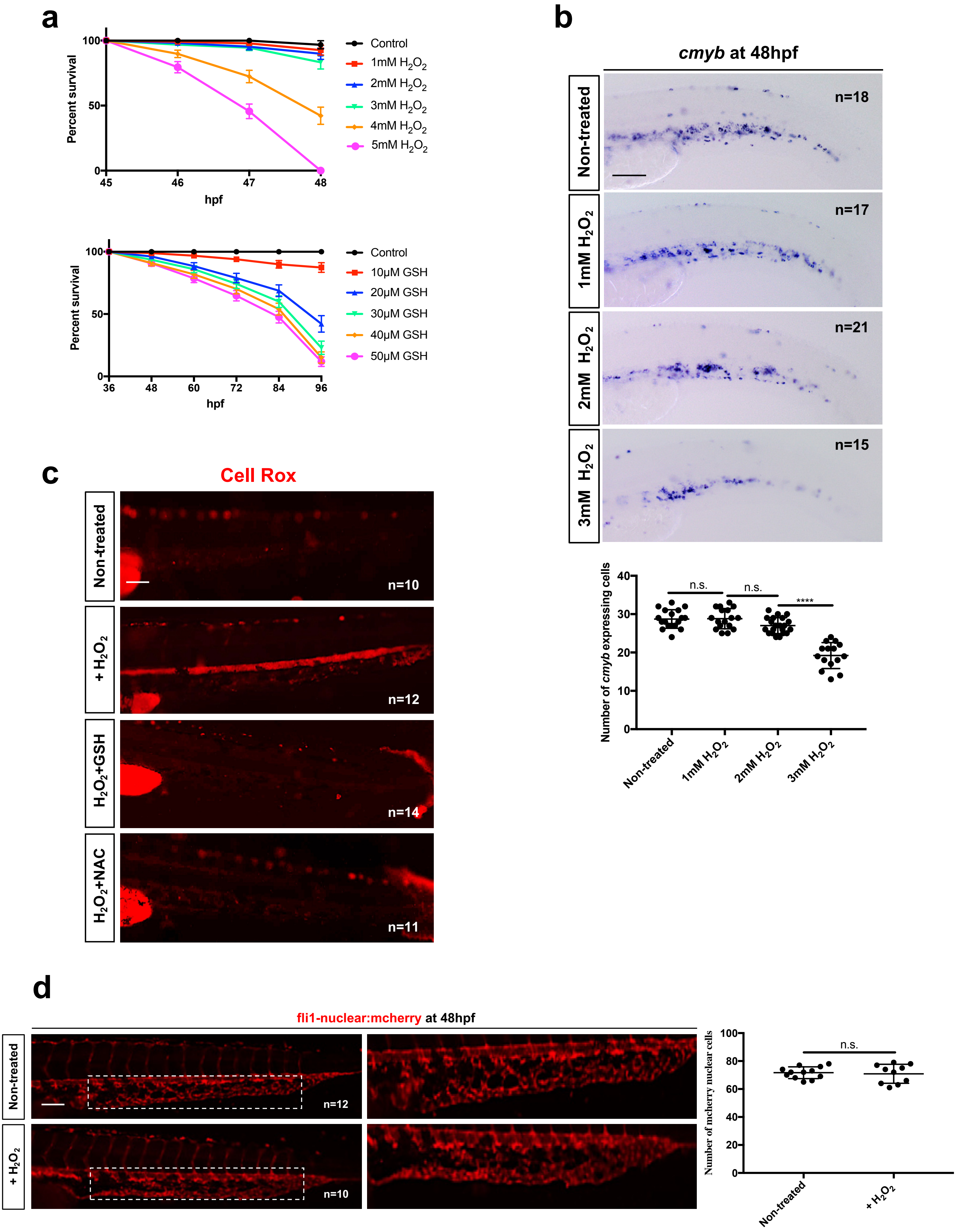

Supplementary Figure 1. H_2O_2 treatment decreases HSPC numbers in the CHT at 48hpf.

(a) Survival of zebrafish embryos exposed for 3 hours (between 45-48hpf) to different concentrations of H_2O_2 and GSH from 36 to 96 hpf. Statistical analysis was completed using Mantel-Cox test (comparison of survival curves), centre values denote the mean, and error values denote s.e.m, ****$P<.0001$. (b) WISH against *cmyb* at 48hpf in NT and after H_2O_2 exposure (1-2-3mM). Statistical analysis was completed using a one-way ANOVA, with Tukey–Kramer post hoc tests, adjusted for multiple comparison, ****$P<.0001$, (n.s.) non-significant $P=0.15$; $P=0.17$, centre values denote the mean, and error values denote s.e.m. (c) Oxidative stress level in zebrafish embryo using a Cell-Rox probe at 48hpf. (d) Fluorescence imaging of *fli1a:nls-mcherry* embryos either non-treated, or after treatment with H_2O_2. Statistical analysis performed using an unpaired two tailed t test, (n.s.) non-significant $P=0.99$. Data are presented as mean values +/- SEM. Scale bar 100µm (b-c-d).

Supplementary figure 2

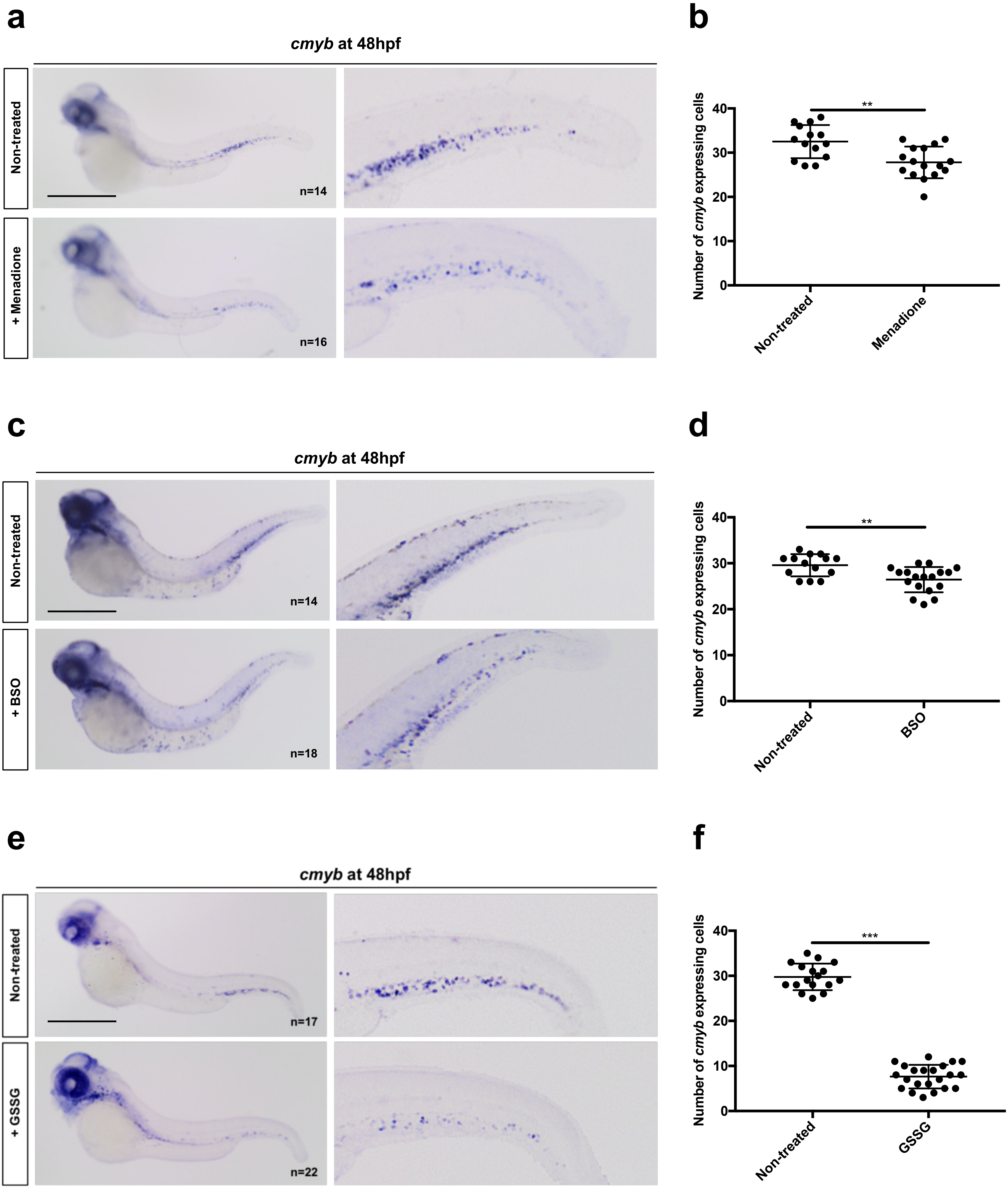

Supplementary Figure 2. Treatments which induce ROS decrease the number of HSPCs in the CHT.

(a) Wish against *cmyb* at 48hpf in non-treated and menadione treated embryos. (b) Quantification of *cmyb*-expressing cells at 48hpf in the CHT. Statistical analysis was performed using an unpaired two tailed t test **P =0.005. Data are presented as mean values +/- SEM. (c) Wish against *cmyb* at 48hpf in non-treated and BSO treated embryos. (d) Quantification of *cmyb*-expressing cells at 48hpf in the CHT. Statistical analysis was performed using an unpaired two tailed t test **P=0.004. Data are presented as mean values +/- SEM. (e) Wish against *cmyb* at 48hpf in non-treated and GSSG treated embryos. (f) Quantification of *cmyb*-expressing cells at 48hpf in the CHT. Statistical analysis was performed using an unpaired two tailed t test ***P <.001. Data are presented as mean values +/- SEM. Scale bar 200μm (a-c-e).

Supplementary figure 3

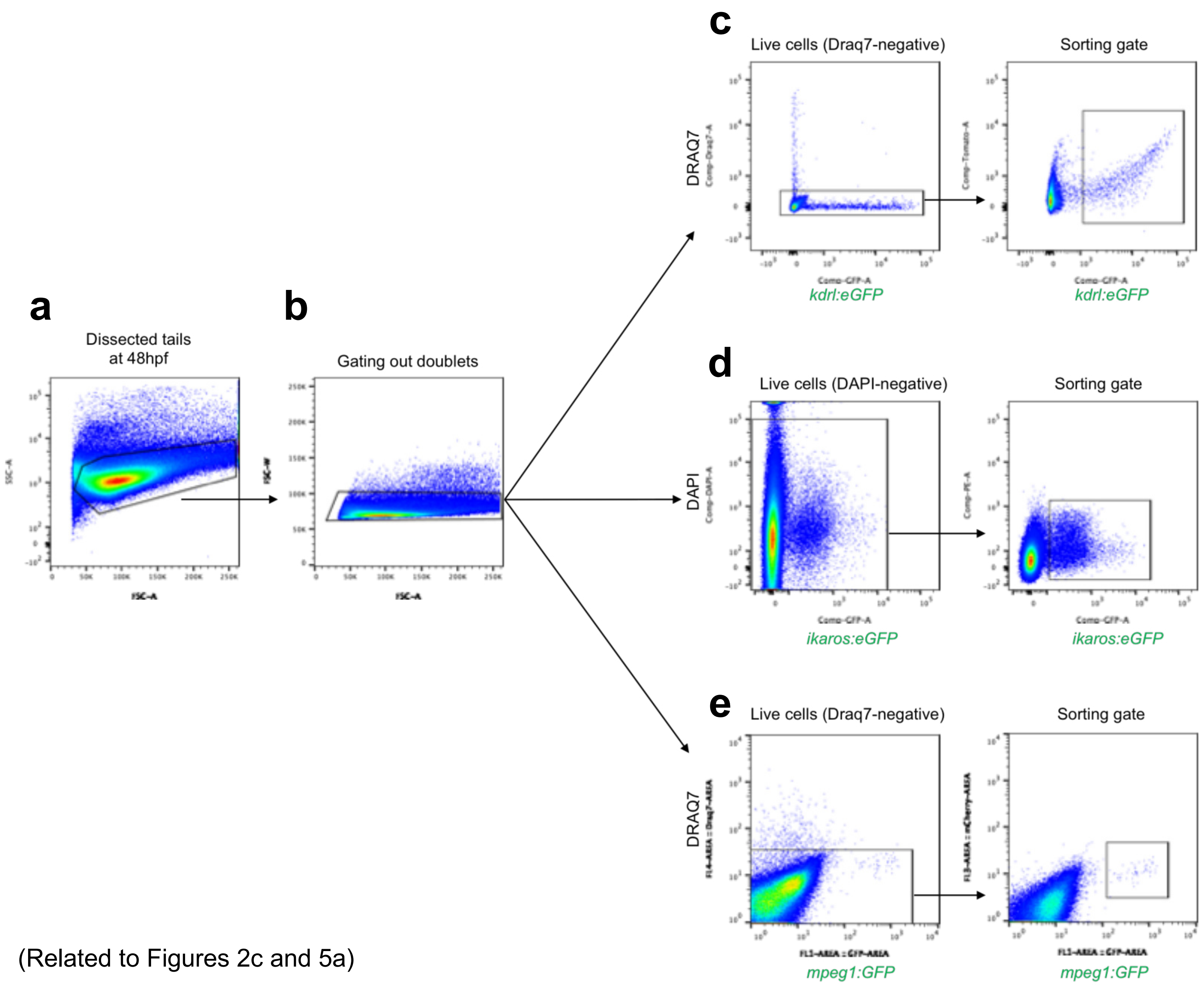

(Related to Figures 2c and 5a)

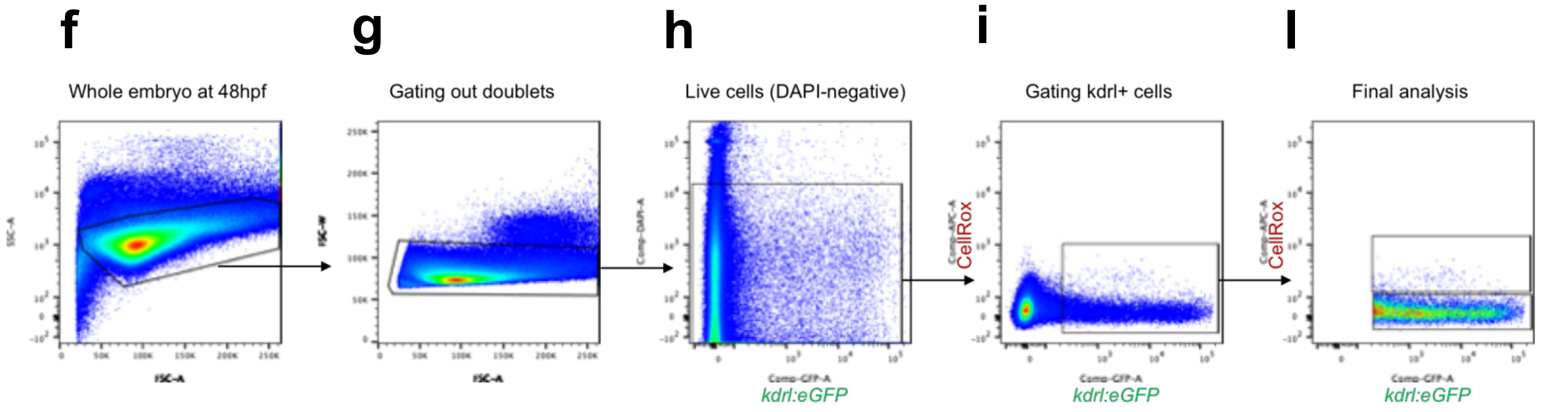

(Related to Figure 4d-g)

Supplementary Figure 3. FACS gating/sorting strategies.

(a-e) To sort endothelial cells (*kdrl:eGFP*), HSPCs (*ikaros:eGFP*) and macrophages (*mpeg1:eGFP*), cell suspensions obtained from dissected tails were submitted to the following gating strategy: (a) using the SSC/FSC plot, we selected a homogeneous population of cells, from which we excluded the doublets (b), by gating on singlets in the FSC-W/SSC-W plot. We then selected live cells, by excluding Draq7 or DAPI positive cells (c-e) and finally sorted *kdrl*, *ikaros*, and *mpeg1*-positive cells according to the gates shown.

(f) To analyse ROS levels in endothelial cells in 48hpf control- and *ifi30*-morphants, we first selected cells based on their SSC/FSC profile. (g) We selected singlets in the FSC-W/SSC-W plot, then selected live cells by gating on DAPI-negative cells (h). Finally, we selected all *kdrl-GFP* positive cells in the CellROX/GFP plot (i), to score the percentage of CellROX-positive versus negative cells in the *kdrl-GFP* population (l).

Supplementary figure 4

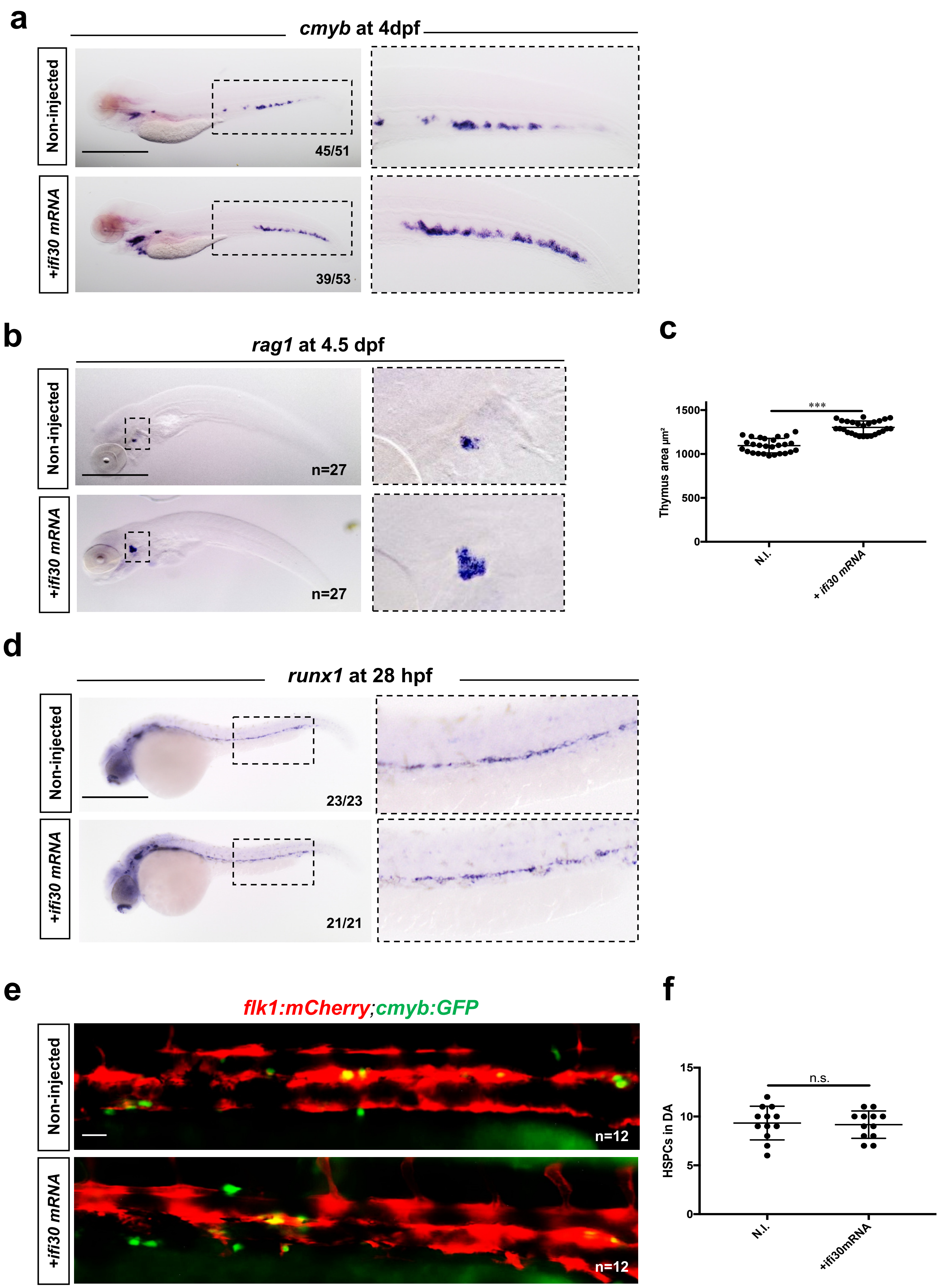

Supplementary Figure 4. *ifi30* overexpression increases the number of HSPCs in the CHT, but does not affect HSPC emergence and specification.

(a) WISH against *cmyb* at 4dpf in non-injected and *ifi30* full-length mRNA injected embryos. Each experiment was repeated independently 3 times with similar results. (b) WISH against *rag1* at 4.5dpf in non-injected and *ifi30* full-length mRNA injected embryos. (c) Quantification of the thymus area. Statistical analysis using an unpaired two tailed t test. ***P < .001. The centre values of all statistical analysis denote the mean, and error values denote s.e.m. (d) WISH against *runx1* at 28hpf in controls and embryos injected with *ifi30* full-length mRNA. Each experiment was repeated independently 3 times with similar results. (e) imaged area in the tail at 36hpf, as indicated by the black dotted line. Confocal imaging in dorsal aorta of double positive *kdrl:mCherry/cmyb:GFP* embryos in non-injected and *ifi30* full-length mRNA injected embryos. White arrows indicate *cmyb*$^{+}$*kdrl*$^{+}$ cells. (f) Quantification of HSPCs closely associated with ECs. Centre values denote the mean, and error values denote s.e.m. statistical analysis was performed using an unpaired two-tailed t test, (n.s.) non-significant P=0.80. Scale bars: 200µm (a-b-d); 50µm (e).

Supplementary figure 5

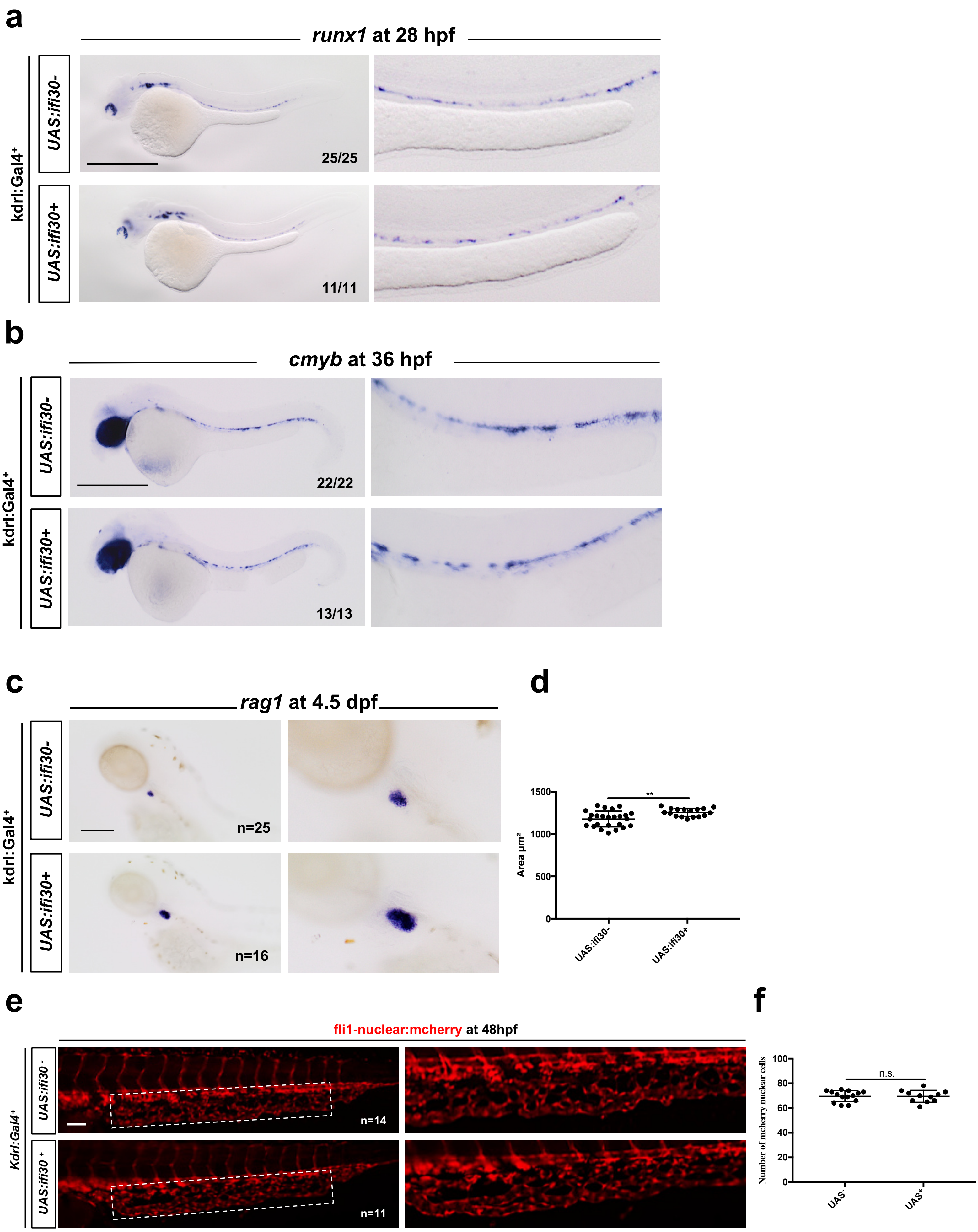

Supplementary Figure 5. *ifi30* controls definitive hematopoiesis at the non-cell-autonomous level in the CHT.

a) WISH for *runx1* at 28 hpf in *kdrl:Gal4*-positive embryos that were either *UAS:ifi30* negative (upper) or positive (lower). Each experiment was repeated independently 3 times with similar results. (b) WISH for *cmyb* at 36hpf in *kdrl:Gal4*-positive embryos that were either *UAS:ifi30* negative (upper) or positive (lower). Each experiment was repeated independently 3 times with similar results. (c) WISH for *rag1* at 4.5dpf in *kdrl:Gal4*-positive embryos that were either *UAS:ifi30* negative (upper) or positive (lower). (d) Quantification of the thymus area. Centre values denote the mean, and error values denote s.e.m. The statistical analysis was performed using an unpaired two-tailed t test, **P=0.005. (e) Fluorescence imaging of *fli1a:nuc-mcherry*/*kdrl:Gal4*-positive embryos that were either *UAS:ifi30* negative (upper) or positive (lower). (f) Quantification of *mcherry* positive cells in the CHT at 48hpf. The statistical analysis was performed using an unpaired two-tailed t test, (n.s.) non-significant P=0.86. Centre values denote the mean, and error values denote s.e.m. Scale bars: 200µm left panel (a-b) and 100µm right panel; 100µm (c-e).

Supplementary figure 6

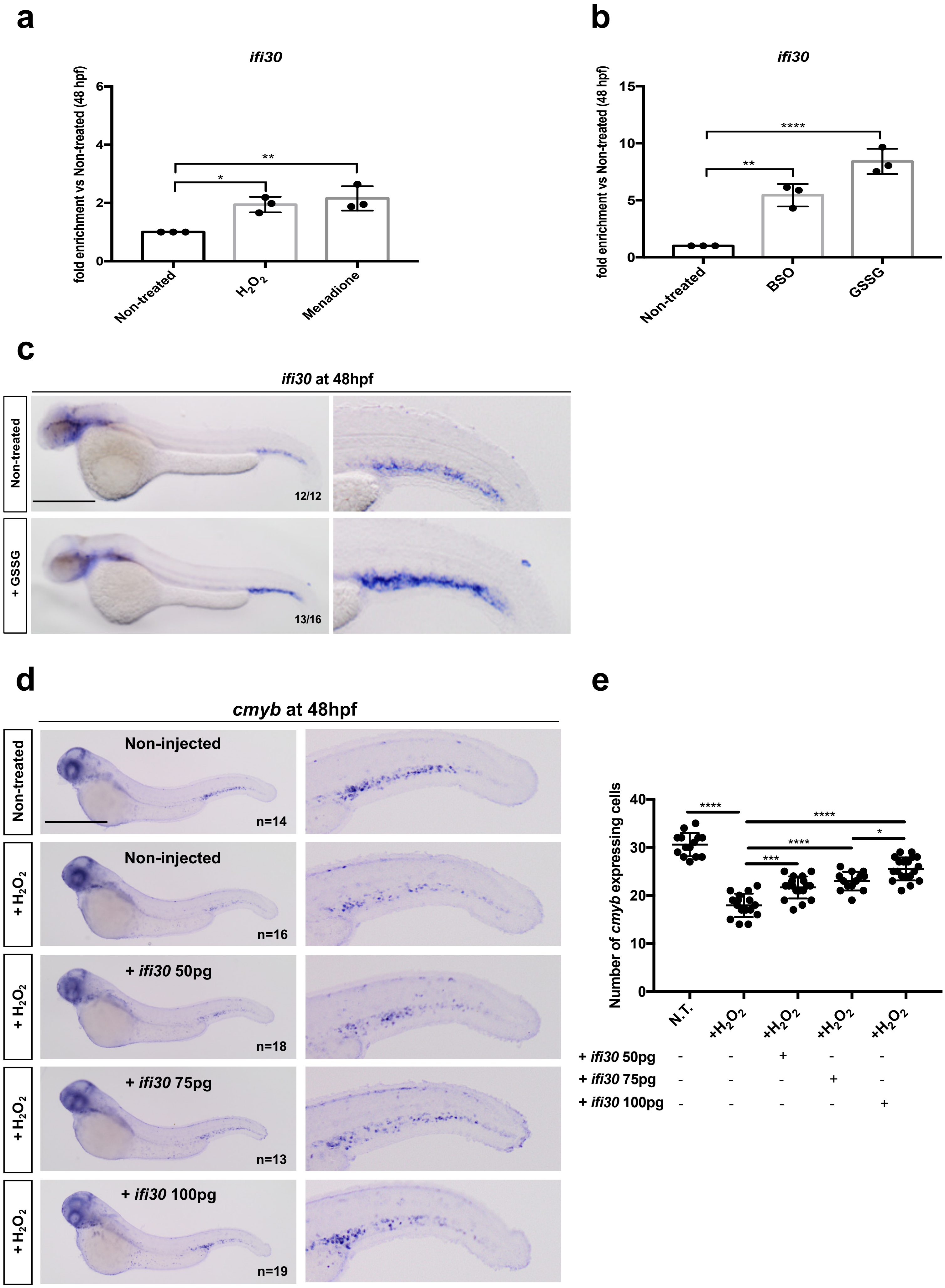

Supplementary Figure 6. The gradual increase in *ifi30* expression rescues the loss HSPCs by oxidative stress.

(a) qPCR for *ifi30* at 48hpf after treatment with H_2O_2 or menadione. The statistical analysis was performed using one-way ANOVA with Tukey–Kramer post hoc tests, adjusted for multiple comparison *P=0.012; **P=0.004. Centre values denote the mean, and error values denote s.e.m, for each condition have been pooled (n=10 animals), and each experiment was repeated independently 3 times. (b) qPCR for *ifi30* at 48hpf after treatments with glutathione synthesis inhibitor buthionine sulfoximine (BSO) and oxidized glutathione GSSG. For each condition have been pooled (n=10 animals), and each experiment was repeated independently 3 times. The statistical analysis was performed using one-way ANOVA with Tukey–Kramer post hoc tests, adjusted for multiple comparison **P=0.0013; ****P<.0001. Centre values denote the mean, and error values denote s.e.m, n=3 independent experiments with biological triplicate. (c) WISH for *ifi30* at 48hpf in non-treated embryos or embryos treated with GSSG. Each experiment was repeated independently 3 times with similar results. (d) WISH for *cmyb* at 48hpf in non-treated and H_2O_2 (3mM) treated embryos injected with *ifi30*-mRNA at different concentrations. (e) Quantification of *cmyb*-expressing cells at 48hpf in the CHT. Statistical analysis was performed using a one-way ANOVA with Tukey–Kramer post hoc tests adjusted for multiple comparison. ****P<.0001; ***P=0.0002; *P=0.049. Centre values denote the mean, and error values denote s.e.m. Scale bars: 200µm (a-b).

Supplementary figure 7

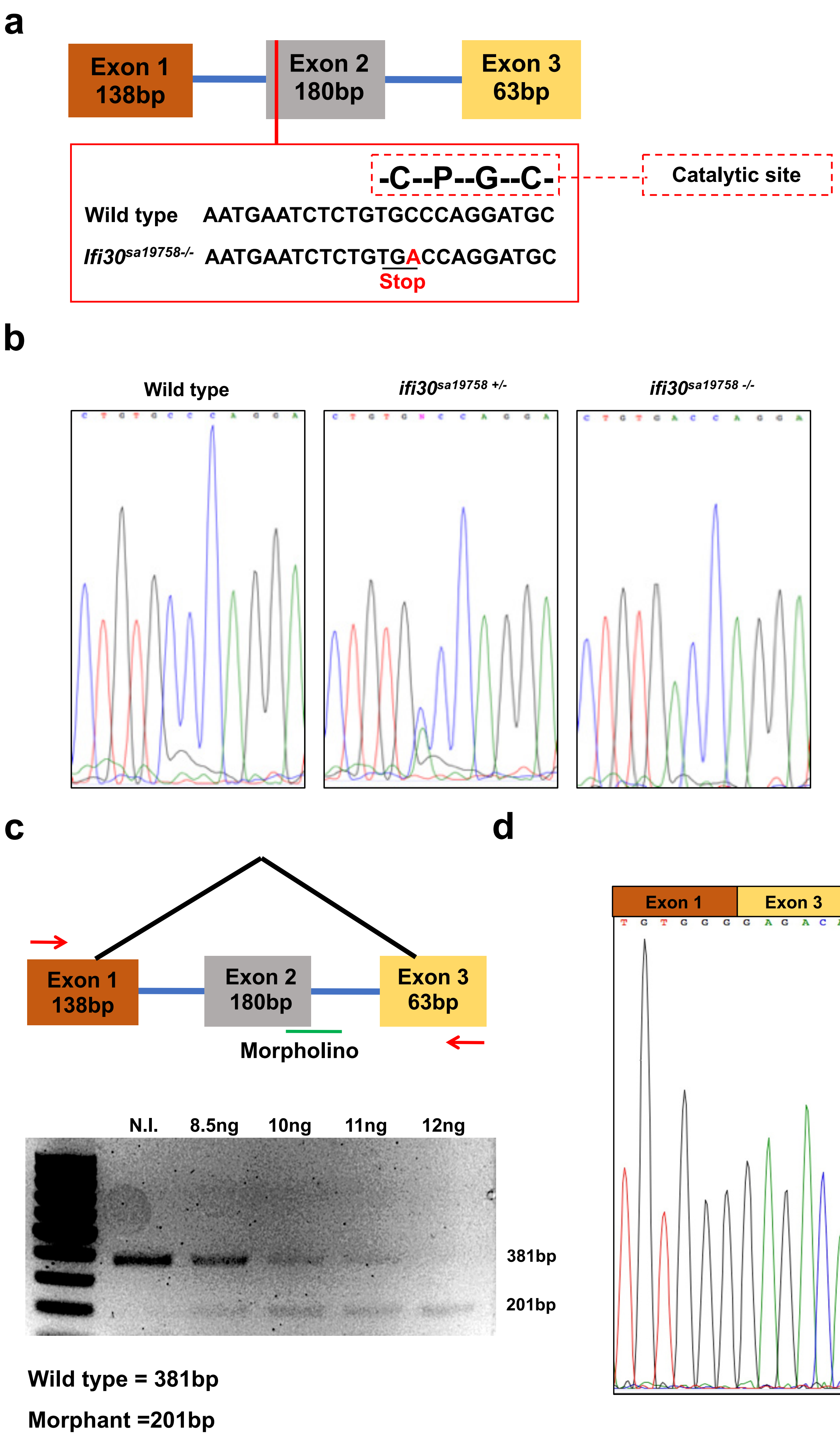

Supplementary Figure 7. The *ifi30* mutation and *ifi30*-morpholino both affect exon2 and the catalytic site.

(a) Schematic of the point-mutation in first cysteine of catalytic site in *ifi30*$^{-/-}$. (b) Sanger-sequencing of PCR product produced when genotyping the *ifi30*^{sa19758} mutant line. (c) Schematic of splice blocking MO targeting intron/exon junctions in *ifi30*. Validation of the *ifi30*-MO which induces exon2 skipping. RT-PCR was performed on mRNA/cDNA obtained at 48hpf from pools of 10 embryos. (d) The PCR products were sequenced using the forward primer.

Supplementary figure 8

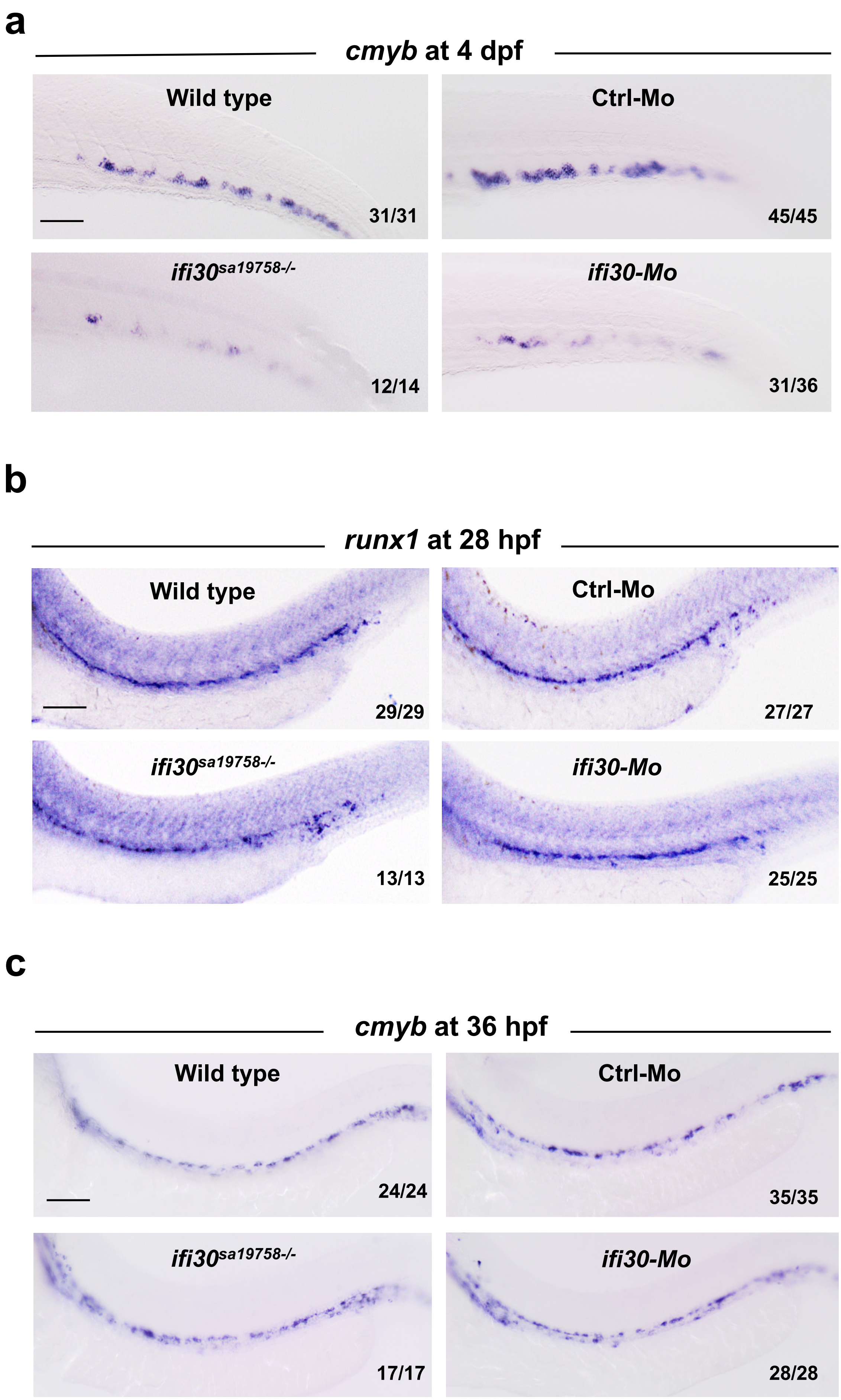

Supplementary Figure 8. ***ifi30*****-deficiency decreases HSPC expansion in the CHT but not HSPC specification.**

(a) WISH for *cmyb* at 4dpf in wild type and *ifi30*$^{-/-}$ after injection with control or *ifi30*-MO. Each experiment was repeated independently 3 times with similar results. (b) WISH for *runx1* at 28hpf in wild type or *ifi30*$^{-/-}$ after injection with control or *ifi30*-MO. Each experiment was repeated independently 3 times with similar results. (c) WISH for *cmyb* at 36 hpf in wild type or *ifi30*$^{-/-}$ after injection with control or *ifi30*-MO. Each experiment was repeated independently 3 times with similar results. Scale bars: 100μm (a-b-c).

Supplementary figure 9

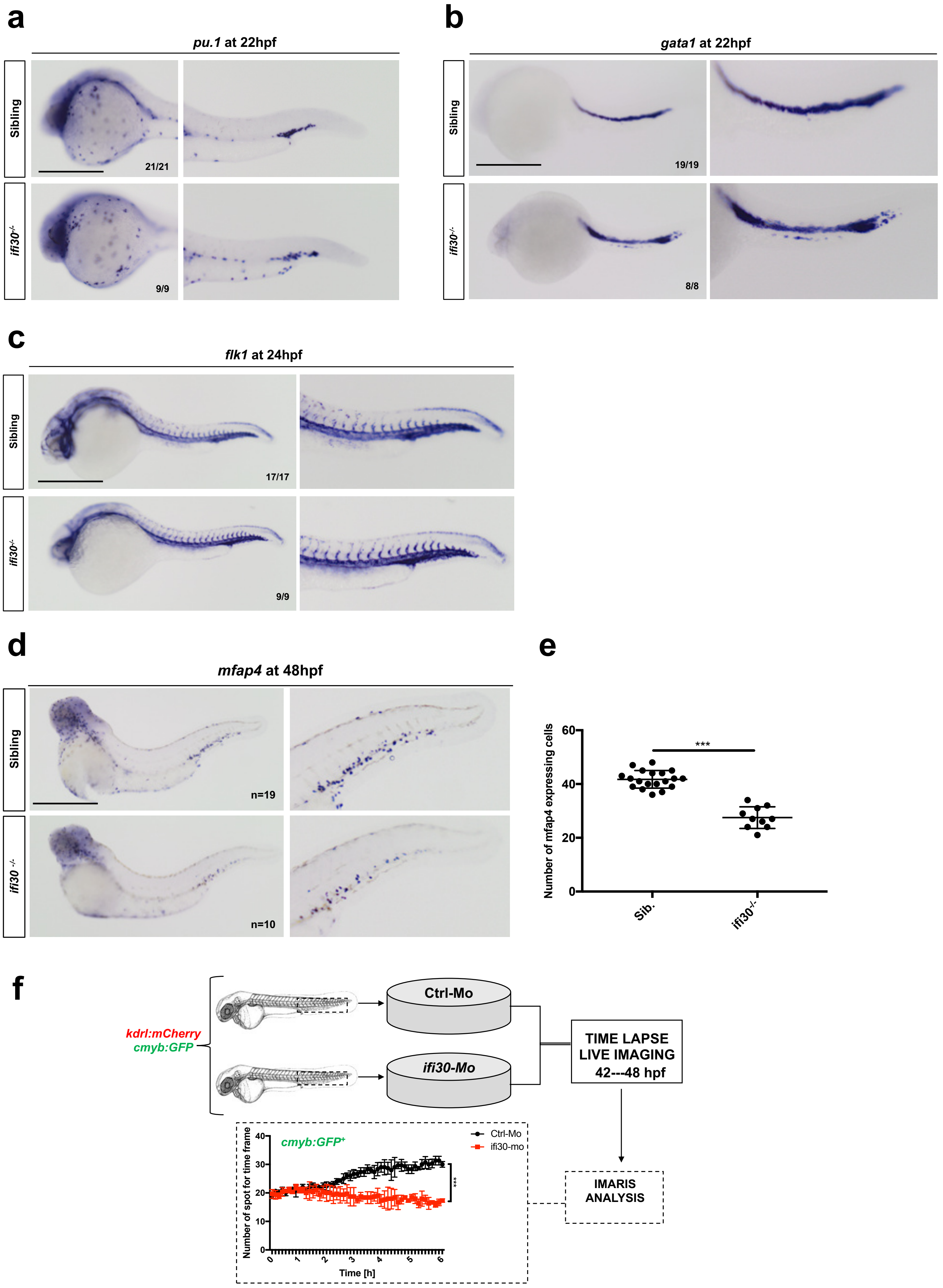

Supplementary Figure 9. *Ifi30*-deficiency does not affect primitive erythropoiesis or myelopoiesis, nor vasculogenesis

(a) WISH for *pu.1* at 22hpf in *ifi30*$^{-/-}$ or sibling. Each experiment was repeated independently 3 times with similar results. (b) WISH for *gata1* at 22hpf *ifi30*$^{-/-}$ or sibling. Each experiment was repeated independently 3 times with similar results. (c) WISH for *flk1* at 24hpf in *ifi30*$^{-/-}$ or sibling. Each experiment was repeated independently 3 times with similar results. (d) WISH for *mfap4* at 48hpf in *ifi30*$^{-/-}$ or sibling. (e) Quantification; statistical analysis was performed using an unpaired two tailed t test. ***P < .001. Centre values denote the mean, and error values denote s.e.m. (f) Experimental outline and quantification of time-lapse live imaging in controls and *ifi30*-morphants *cmyb:GFP*$^{+}$ cells, performed using Imaris software. For each condition have been used (n=9 animals), and each experiment was repeated independently 3 times, with similar results. Centre values denote the mean, and error values denote s.e.m. The statistical analysis was performed using an unpaired two-tailed t test. ***P < .001. Scale bars: 200µm (a-b-c-d).

Supplementary figure 10

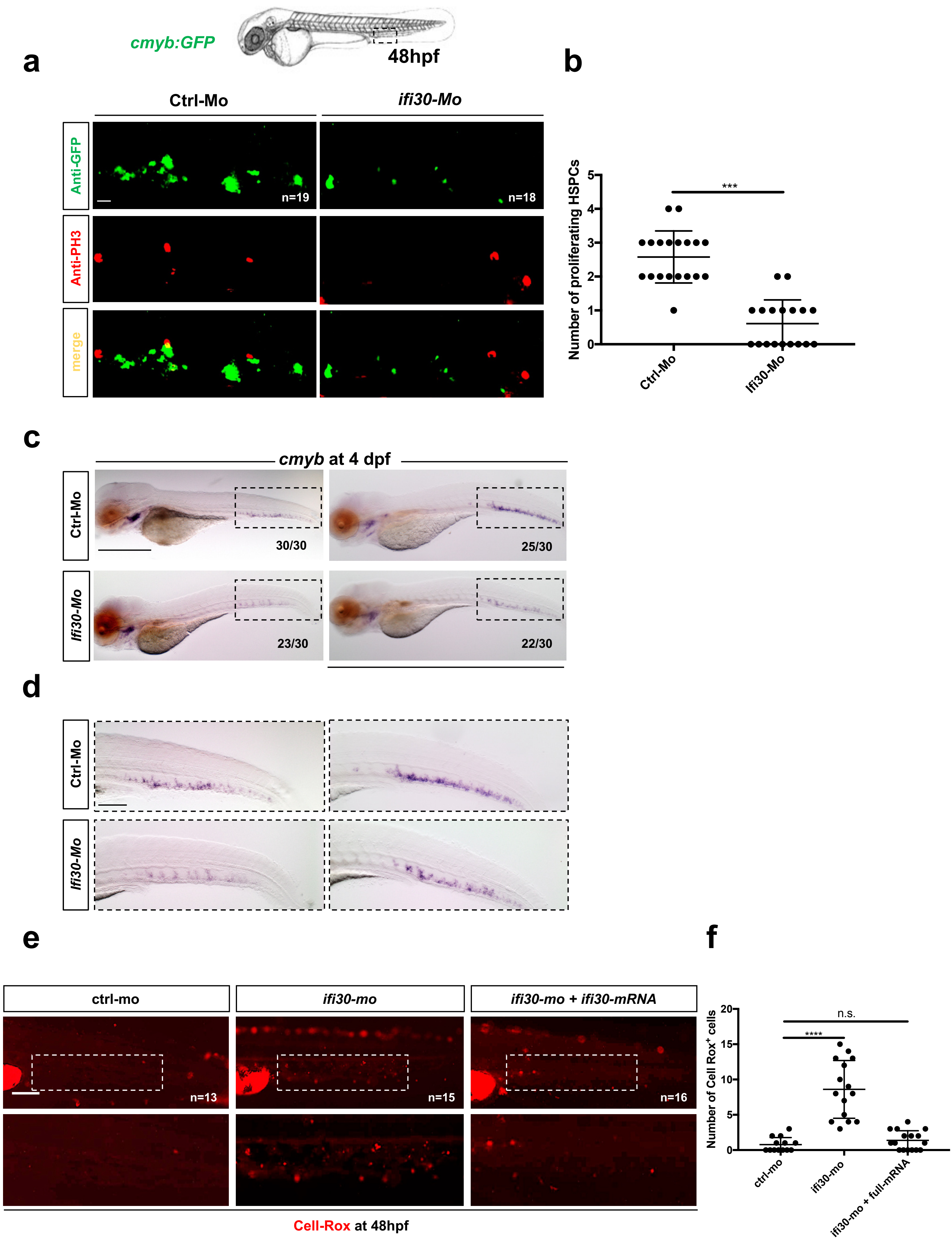

Supplementary Figure 10. *ifi30*-morphants present a defect in HSPC proliferation.

(a) Anti-GFP and pH3 immunostainings of either control (Ctrl-Mo) or *ifi30*-morphant *(ifi30-MO*) *cmyb:GFP* embryos. (b) Quantification of the number of the pH3+ HSPCs in controls or *ifi30*-morphants. Centre values denote the mean, and error values denote s.e.m, statistical analysis was performed using an unpaired two-tailed t test, ***P<.001. (c) WISH for *cmyb* at 4dpf after the injection of control-MO, *ifi30*-MO or *ifi30*-full mRNA. Each experiment was repeated independently 3 times with similar results. (d) Magnification of the tail region, as indicated by the black dotted line. Each experiment was repeated independently 3 times with similar results. (e) Fluorescence imaging to detect the Cell-Rox fluorescent probe (red) in the CHT of AB* embryos at 48 hpf after the injection of control-MO, *ifi30*-MO or *ifi30*-full mRNA. (f) Quantification of the number of Cell-ROX positive cells, Statistical analysis was performed using a one-way ANOVA with Tukey–Kramer post hoc tests, adjusted for multiple comparison ****P<.0001, (n.s.) non-significant P=0.80. Centre values denote the mean, and error values denote s.e.m. Scale bars: 25µm (a), 200µm (c), 100µm (d-e).

Supplementary figure 11

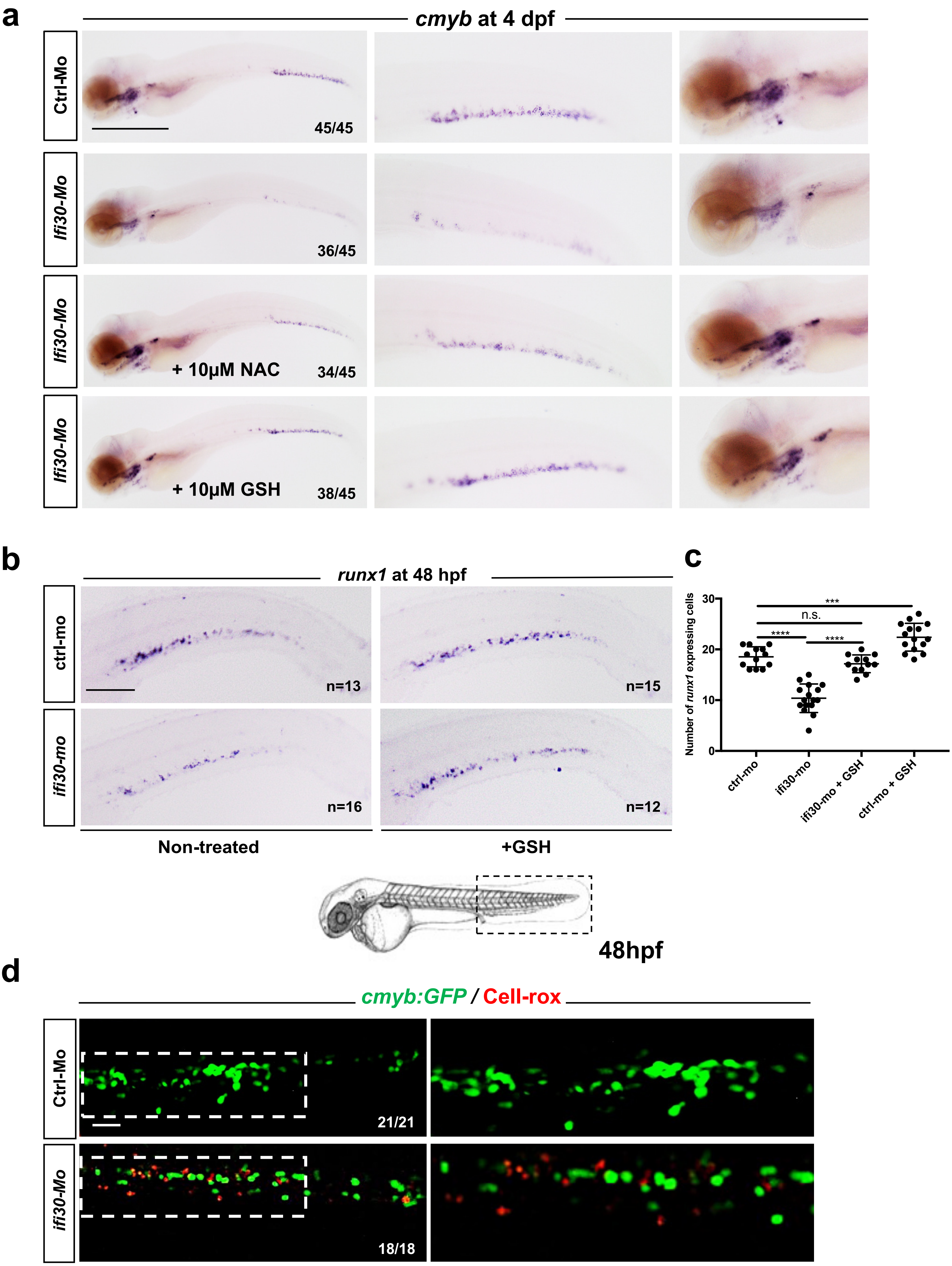

Supplementary Figure 11. The loss of HSPCs in *ifi30*-morphants is rescued by antioxidant treatments.

(a) WISH for *cmyb* at 4dpf after the injection with control-MO or *ifi30*-MO after treatment with NAC (N-acetyl-cysteine) or GSH (reduced-glutathione). Each experiment was repeated independently 3 times with similar results. (b) WISH for *runx1* at 48hpf after injection with control-MO or *ifi30*-MO and after treatment with GSH (reduced-glutathione). Each experiment was repeated independently 3 times with similar results. (c) Statistical analysis was performed using a one-way ANOVA with Tukey–Kramer post hoc tests multiple comparison, ****P<.0001; ***P=0.0006; (n.s.) non-significant P=0.49. Centre values denote the mean, and error values denote s.e.m. (d) Imaged region in the tail at 48hpf, as indicated by the black dotted line. Confocal imaging of the CHT of *cmyb:GFP* (green) and Cell-Rox fluorescent probe (red) in control (upper) or *ifi30*-morphants (lower). The left panel is a magnification of the region delimited by the white dotted line. Each experiment was repeated independently 3 times with similar results. Scale bars: 200µm (a); 100µm (b); 50µm (d).

Supplementary figure 12

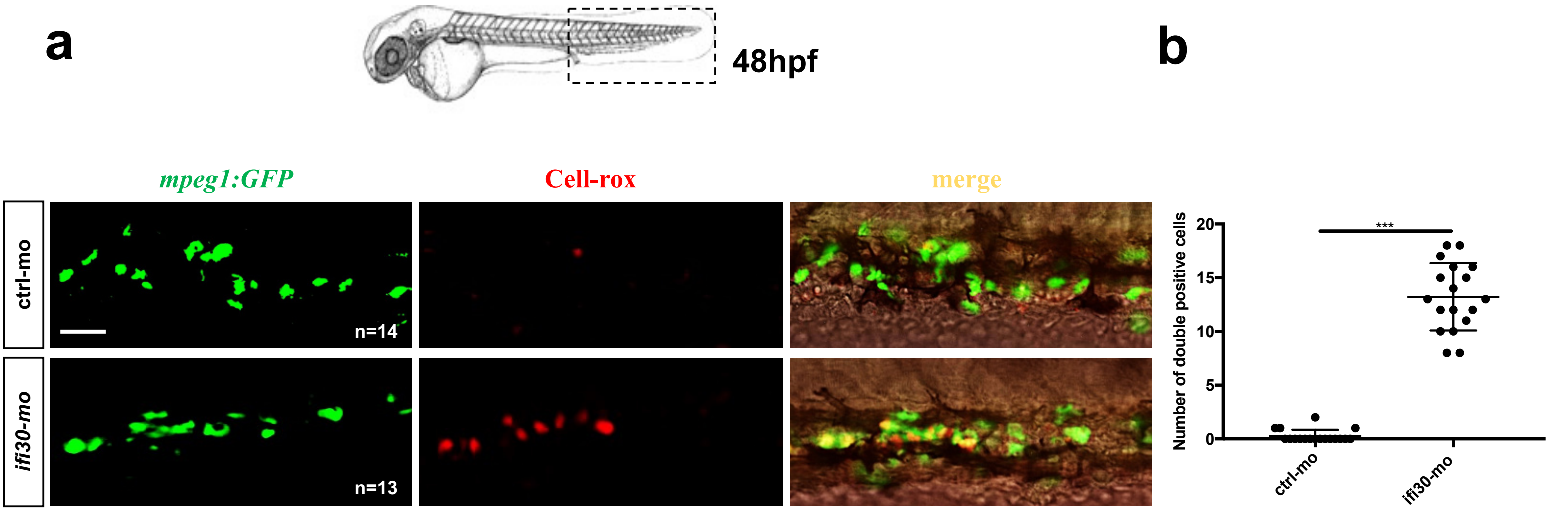

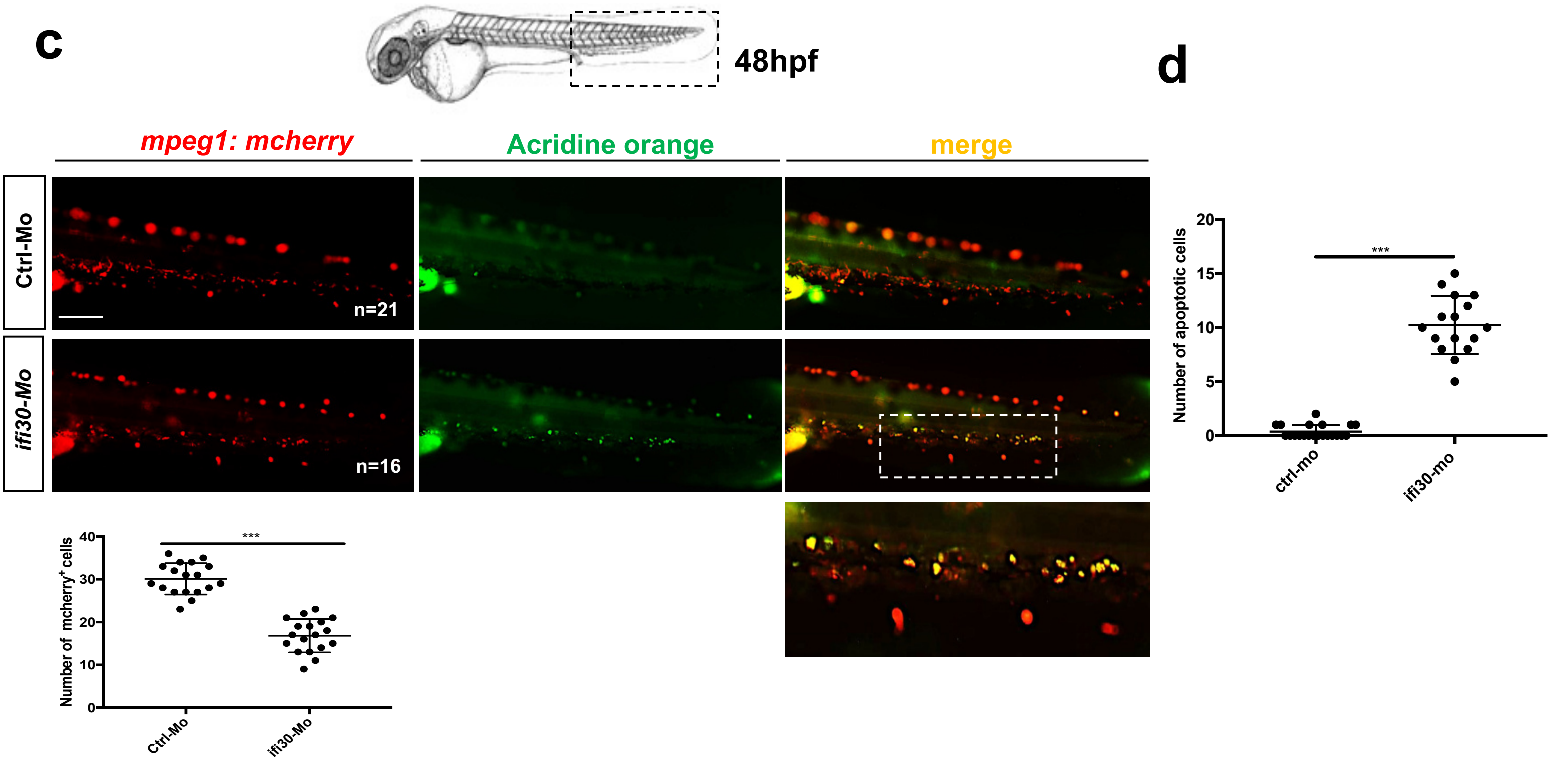

Supplementary Figure 12. *ifi30*-morphants show an increase in ROS in macrophages, inducing their death.

(a) Confocal imaging of the CHT of *mpeg1:GFP* (green), Cell-Rox fluorescent probe (red) and merge (yellow), in control-MO or *ifi30*-MO injected embryos. (b) The graph represents the quantification of double positive cells in control-MO and *ifi30*-MO injected embryos. Statistical analysis was performed using an unpaired two-tailed t test. ***P<.001. Centre values denote the mean, and error values denote s.e.m. (c) Imaged area in the tail at 48hpf (black dotted line). Fluorescence imaging of the CHT of *mpeg1:mcherry* (red), acridine orange (green) and merge (yellow), in control-MO or *ifi30*-MO injected embryos. The graph represents the quantification of *mpeg1:mcherry*$^+$ in controls and *ifi30*-morphants. Centre values denote the mean, and error values denote s.e.m. Statistical analysis was performed using an unpaired two-tailed t test. ***P<.001. (d) The graph represents the quantification of apoptotic cells in controls and *ifi30*-morphants. Centre values denote the mean, and error values denote s.e.m. Statistical analysis was performed using an unpaired two-tailed t test. ***P<.001. Scale bars: 50µm (a); 100µm (c).

Supplementary figure 13

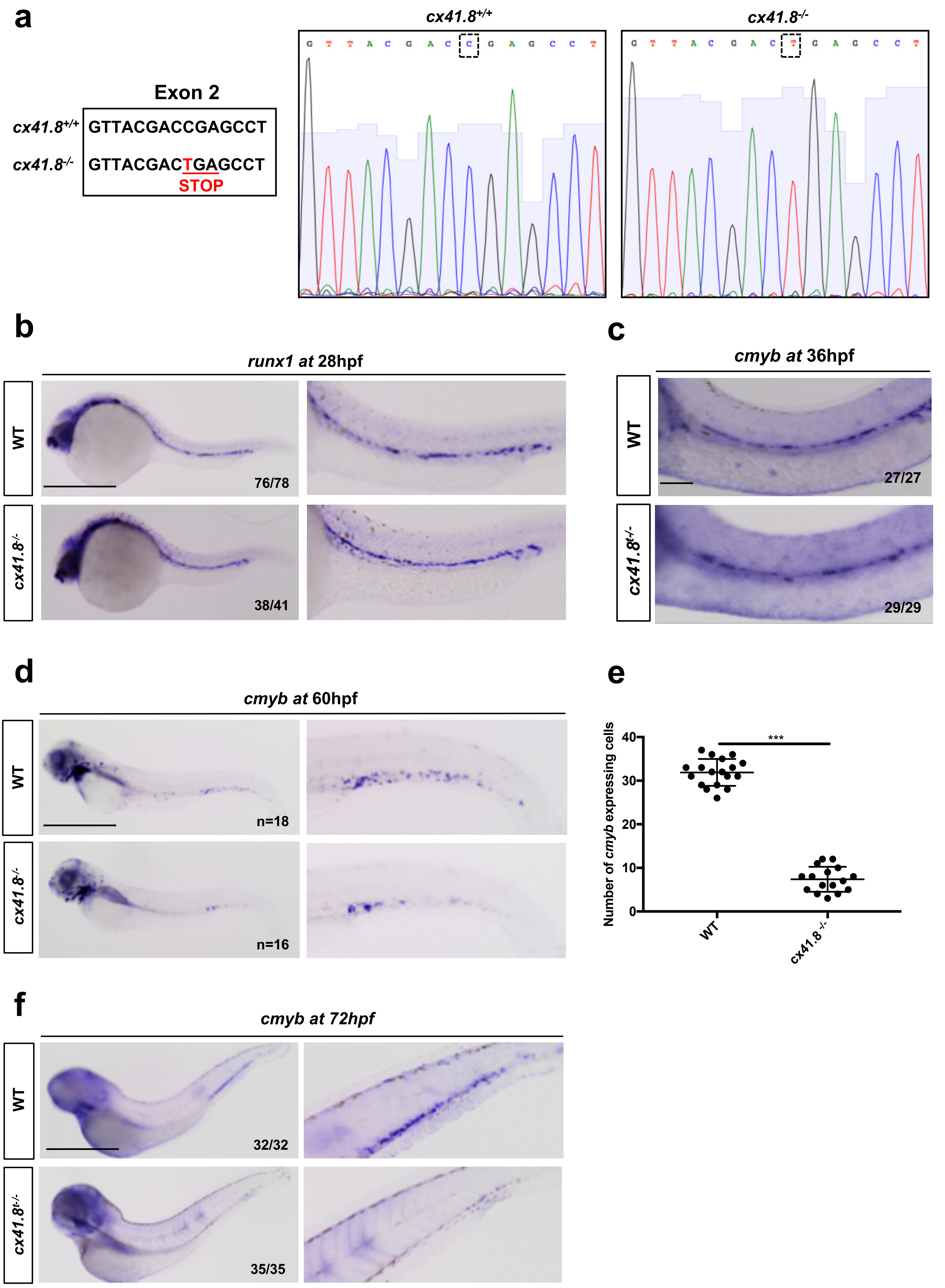

Supplementary Figure 13. The connexin mutant *cx41.8*$^{-/-}$ presents an HSPCs expansion defect in the CHT at 60hpf.

(a) Schematic of the point-mutation in *cx41.8*$^{-/-}$ compared with wild type. Sanger-sequencing of PCR product produced when genotyping the *cx41.8*$^{-/-}$ mutant line. (b) In situ hybridization for *runx1* at 28hpf in wild type and *cx41.8*$^{-/-}$embryos. Each experiment was repeated independently 3 times with similar results. (c) In situ hybridization for *cmyb* at 36hpf in wild type and *cx41.8*$^{-/-}$ embryos. Each experiment was repeated independently 3 times with similar results. (d) In situ hybridization for *cmyb* at 60hpf in wild type and *cx41.8*$^{-/-}$ embryos. (e) Quantification of *cmyb*-expressing cells in the CHT. Statistical analysis was performed using an unpaired two-tailed t test. ***P < .001. Centre values denote the mean, and error values denote s.e.m. (f) In situ hybridization for *cmyb* at 72hpf in wild type and *cx41.8*$^{-/-}$embryos. Each experiment was repeated independently 3 times with similar results. Scale bar 200µm (b-d); 100µm (c-f).

Supplementary figure 14

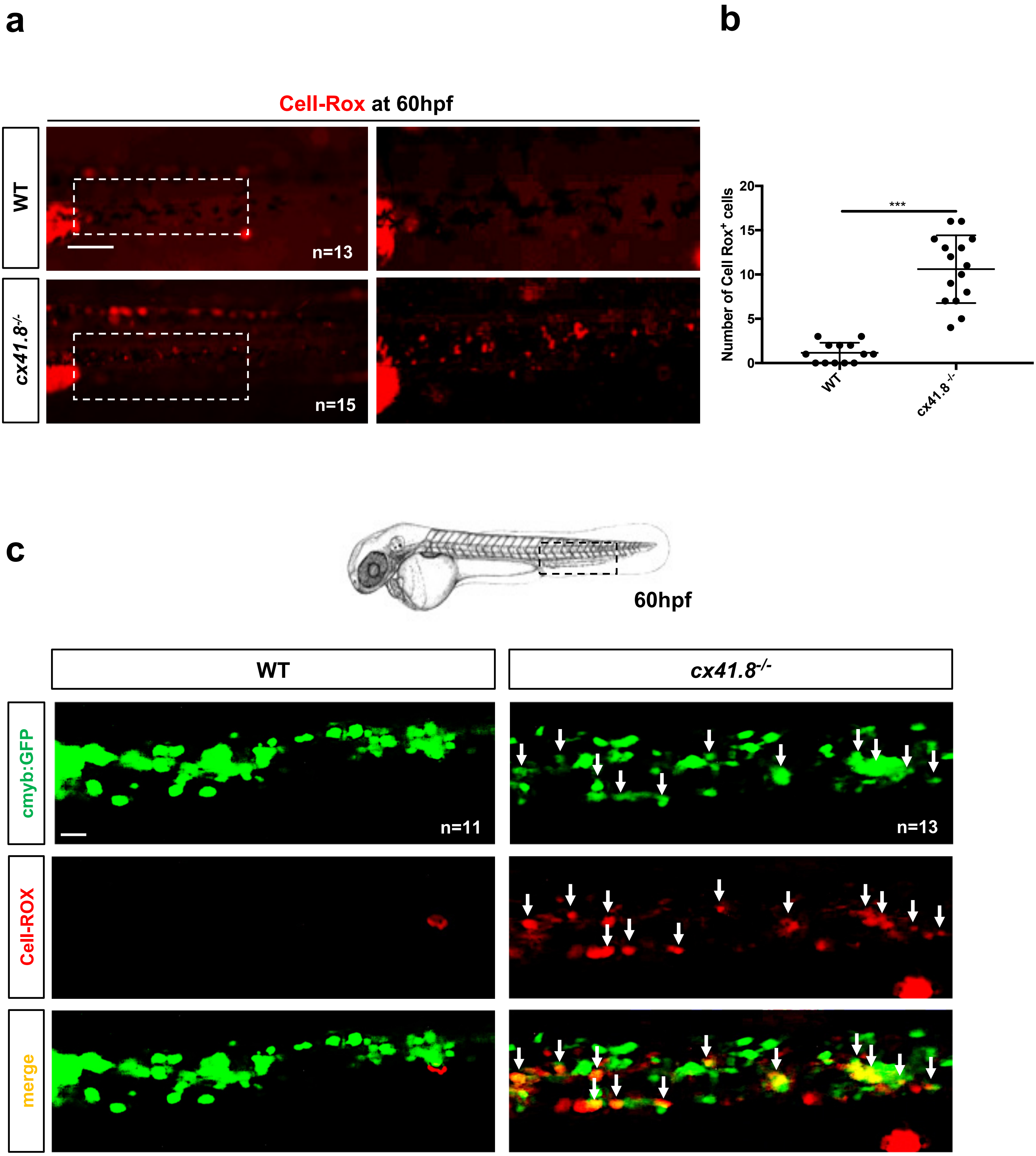

Supplementary Figure 14. The connexin mutant *cx41.8*$^{-/-}$ presents an increase of ROS in HSPCs at 60hpf.

(a) Cell-Rox fluorescent probe detection in wild type and *cx41.8*$^{-/-}$ embryos. (b) Quantification of Cell-Rox positive cells. Statistical analysis was performed using an unpaired two-tailed t test. ***P<.001. Centre values denote the mean, and error values denote s.e.m. (c) Cell-Rox fluorescent probe detection in wild type and *cx41.8*$^{-/-}$;cmyb:GFP^{+} embryos. Scale bar 100μm (a); 50μm (c).

Supplementary figure 15

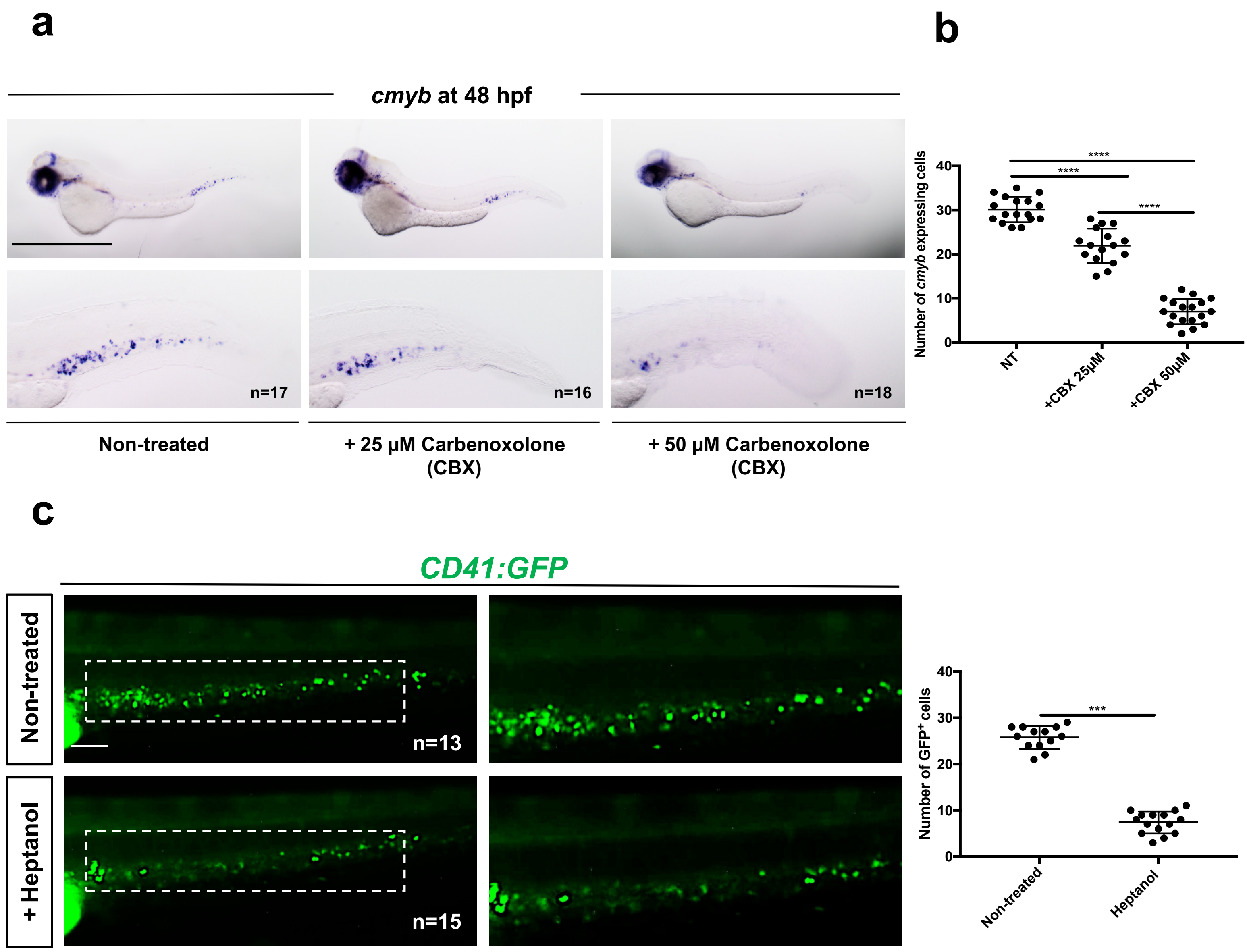

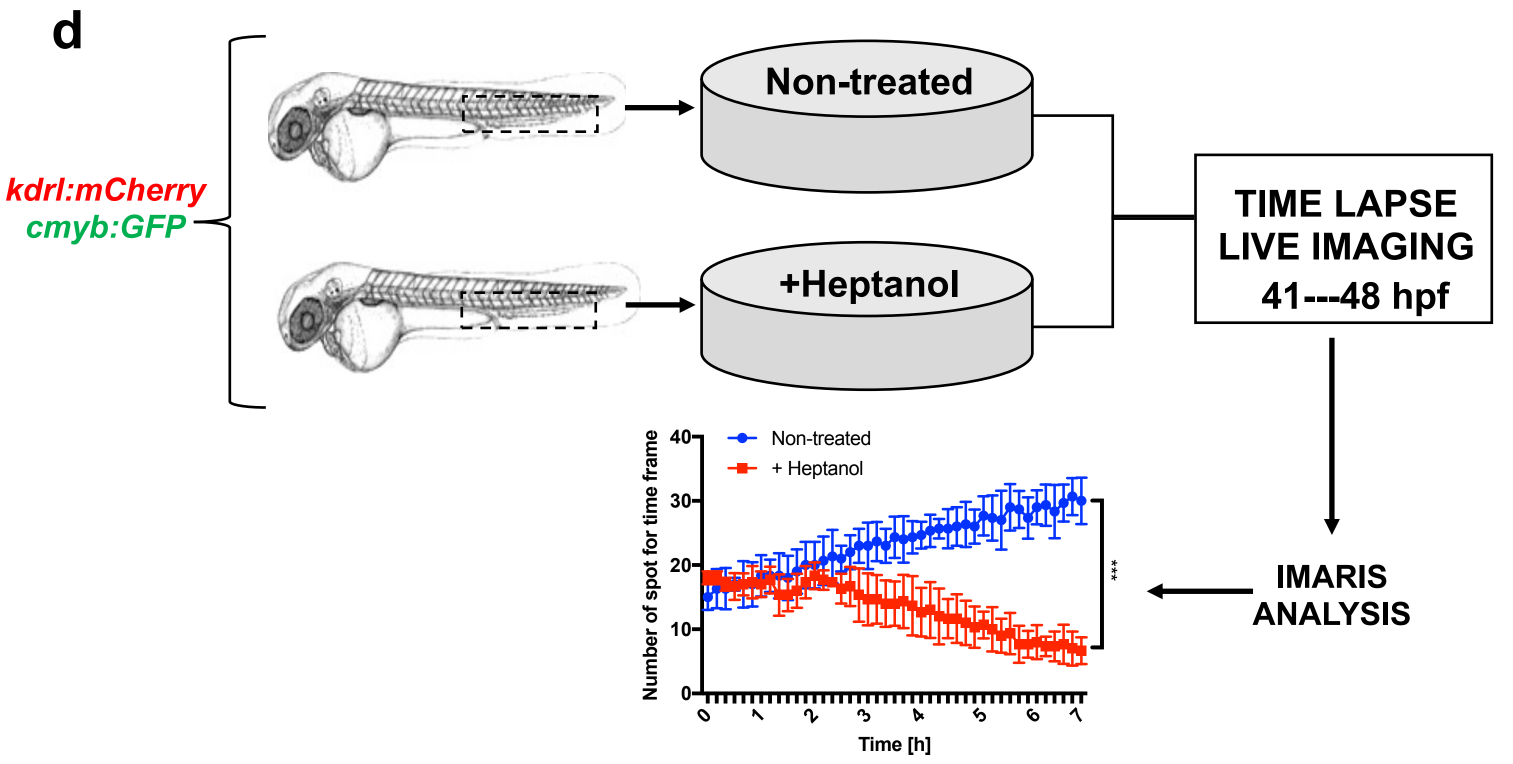

Supplementary Figure 15. Connexin inhibition decreases the number of HSPCs in the CHT at 48hpf.

(a) WISH against *cmyb* at 48hpf in NT embryos or embryos treated with carbenoxolone (25µM and 50µM). (b) Quantification of *cmyb*-expressing cells in the CHT at 48hpf. Statistical analysis was performed using a one-way ANOVA with Tukey–Kramer post hoc tests, multiple comparisons ****P < .0001. Centre values denote the mean, and error values denote s.e.m. (c) Imaged area in the tail at 48hpf of *CD41:GFP* embryos as indicated by the black dotted line in non-treated or heptanol-treated embryos. Quantification of *CD41:GFP*$^{+}$ cells. Statistical analysis was performed using an unpaired two-tailed t test. ***P < .001. Centre values denote the mean, and error values denote s.e.m. (d) Experimental outline of time-lapse live imaging of *GFP*$^{+}$ cells in non-treated and heptanol-treated *cmyb:GFP* embryos. For each condition have been used (n=9 animals), and each experiment was repeated independently 3 times. Quantification was carried out using Imaris software. Statistical analysis was performed using an unpaired two-tailed t test. ***P < .001. Centre values denote the mean, and error values denote s.e.m.. Scale bars: 200µm (a); 100µm (c).

Supplementary figure 16

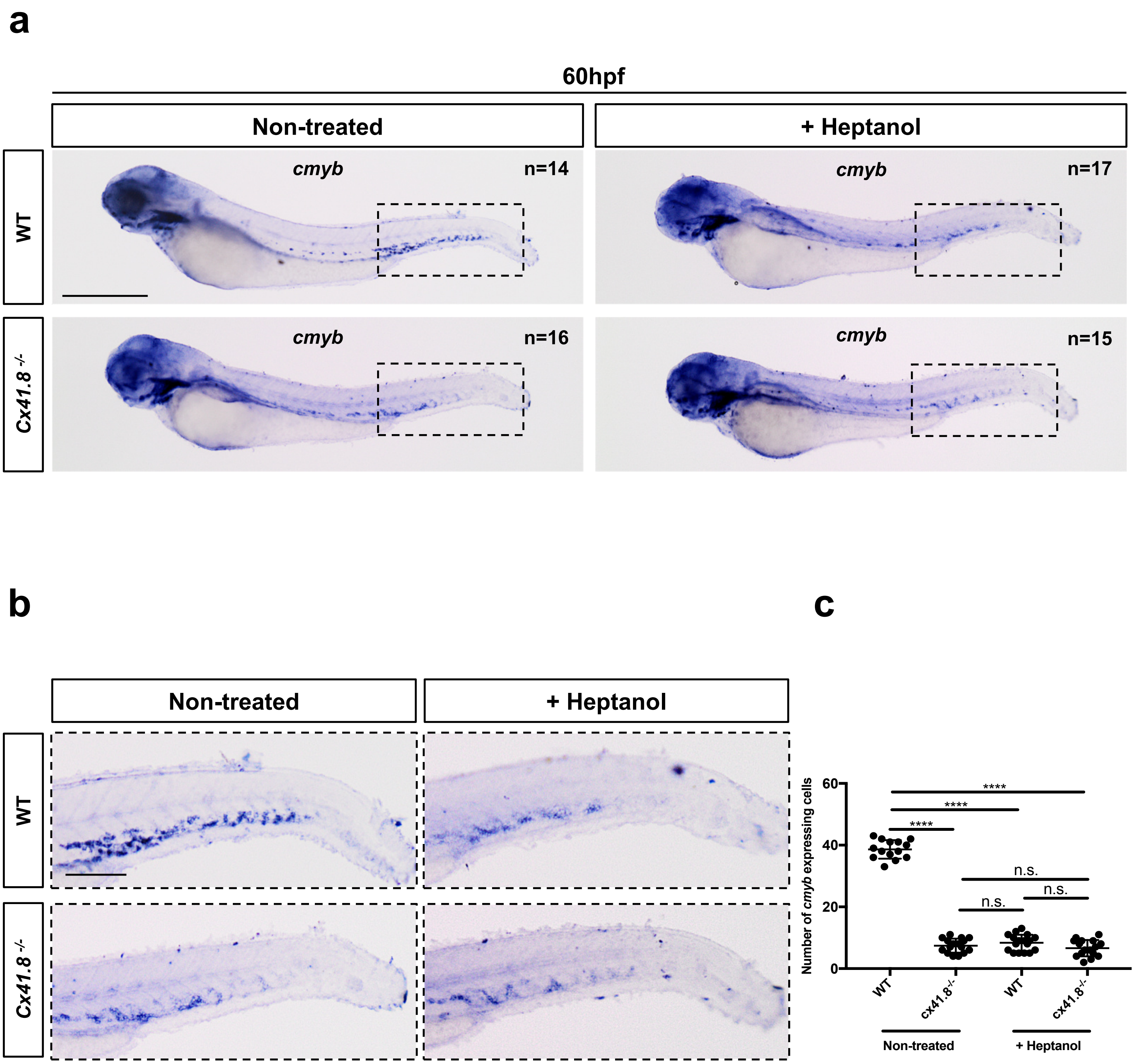

Supplementary Figure 16. The cx41.8 mutant phenocopies connexin inhibition by heptanol treatment.

(a) WISH for *cmyb* at 60hpf in wild type and cx41.8$^{-/-}$ non-treated and after heptanol treatment. (b) Zoom of CHT region to score the number of cmyb expressing cells in wild type and cx41.8$^{-/-}$ non-treated and after heptanol treatment. Each experiment was repeated independently 3 times with similar results. (c) Quantification of *cmyb*-expressing cells in the CHT at 48hpf. Statistical analysis was performed using a one-way ANOVA with Tukey–Kramer post hoc tests adjusted for multiple comparisons ****P<.0001; (n.s.) non-significant P=0.75; P=0.82; P=0.24. Centre values denote the mean, and error values denote s.e.m. Scale bars: 200μm (a); 100μm (b)

Supplementary figure 17

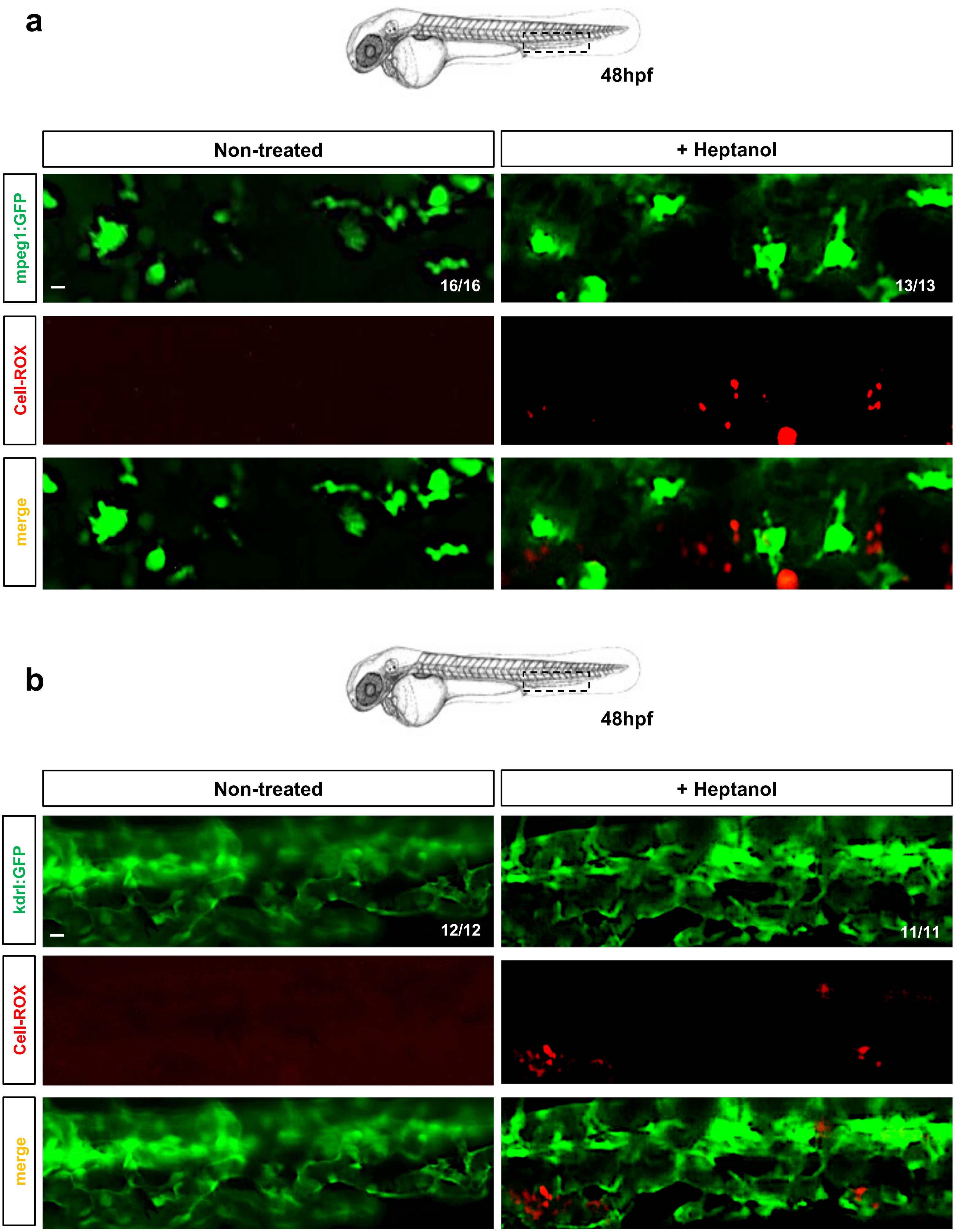

Supplementary Figure 17. Connexin inhibition by heptanol does not induce ROS accumulation in macrophage or ECs.

(a) Cell-ROX detection in the CHT region of non-treated or heptanol-treated *mpeg1:GFP* embryos. Each experiment was repeated independently 3 times with similar results (b) Cell-ROX detection in the CHT region of non-treated or heptanol-treated *kdrl:GFP* embryos. Each experiment was repeated independently 3 times with similar results Scale bars: 50µm (a-b).

Supplementary figure 18

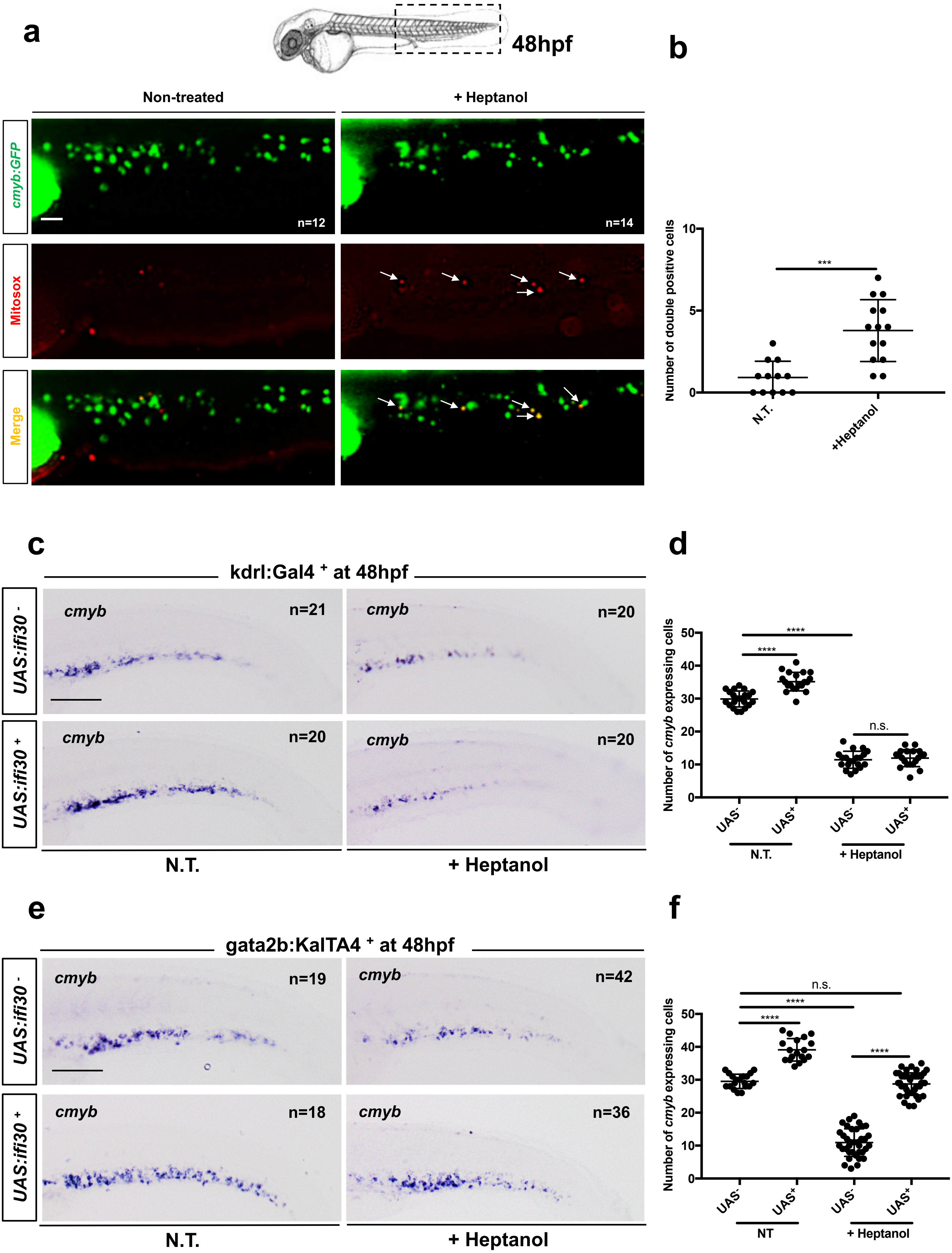

Supplementary Figure 18. The connexin deficiency induces ROS accumulation in HSPCs, causing their cell death, but can be rescued by overexpression of *ifi30*.

(a) Mitosox-Red detection (to identify mitochondrial stress) in the CHT region of non-treated or heptanol-treated *cmyb:GFP* embryos. (b) Quantification of double-positive cells. Centre values denote the mean, and error values denote s.e.m. Statistical analysis was performed using an unpaired-two tailed t test. ***P<.001. (c) *cmyb* expression at 48hpf in *kdrl:Gal4*$^{+}$ embryos which were either *UAS:ifi30*$^{-}$ (upper) or *UAS:ifi30*$^{+}$ (lower), N.T. or heptanol-treated. (d) Quantification of *cmyb*-expressing cells. Statistical analysis: one-way ANOVA, with Tukey–Kramer post hoc tests multiple comparisons. ****P < .0001; (n.s.) non-significant P=0.98. The centre values of all statistical analyses denote the mean and error values denote s.e.m. (e) *cmyb* expression at 48hpf in *gata2b:KalTA4*$^{+}$ embryos which were either *UAS:ifi30*$^{-}$ (upper) or *UAS:ifi30*$^{+}$ (lower), N.T. and heptanol-treated. (f) Quantification of *cmyb*-expressing cells. Statistical analysis: one-way ANOVA, with Tukey–Kramer post hoc tests multiple comparisons. ****P< .0001, (n.s.) non-significant P=0.84. Centre values denote the mean, and error values denote s.e.m. Scale bar: 50μm (a); 100μm (c-e).
