# Supplementary material for: A connexin/ifi30 pathway bridges HSCs with their niche to dampen oxidative stress
Source: Nat Commun. 2021 Jul 23;12:4484. doi: 10.1038/s41467-021-24831-0 (PMC8302694; doi:10.1038/s41467-021-24831-0)
Supplement: Supplementary file 3 — Reporting Summary [file 41467_2021_24831_MOESM3_ESM.pdf]

nature research

Corresponding author(s): Julien Y. Bertrand, revised version NCOMMS-20-01601E

Last updated by author(s): 31/05/21

Reporting Summary

Nature Research wishes to improve the reproducibility of the work that we publish. This form provides structure for consistency and transparency in reporting. For further information on Nature Research policies, see our Editorial Policies and the Editorial Policy Checklist.

Statistics

For all statistical analyses, confirm that the following items are present in the figure legend, table legend, main text, or Methods section.

n/a	Confirmed	
☐	☒	The exact sample size (n) for each experimental group/condition, given as a discrete number and unit of measurement
☐	☒	A statement on whether measurements were taken from distinct samples or whether the same sample was measured repeatedly
☐	☒	The statistical test(s) used AND whether they are one- or two-sided<br>*Only common tests should be described solely by name; describe more complex techniques in the Methods section.*
☐	☒	A description of all covariates tested
☐	☒	A description of any assumptions or corrections, such as tests of normality and adjustment for multiple comparisons
☐	☒	A full description of the statistical parameters including central tendency (e.g. means) or other basic estimates (e.g. regression coefficient) AND variation (e.g. standard deviation) or associated estimates of uncertainty (e.g. confidence intervals)
☐	☒	For null hypothesis testing, the test statistic (e.g. F, t, r) with confidence intervals, effect sizes, degrees of freedom and P value noted<br>*Give P values as exact values whenever suitable.*
☒	☐	For Bayesian analysis, information on the choice of priors and Markov chain Monte Carlo settings
☒	☐	For hierarchical and complex designs, identification of the appropriate level for tests and full reporting of outcomes
☒	☐	Estimates of effect sizes (e.g. Cohen's d, Pearson's r), indicating how they were calculated

Our web collection on statistics for biologists contains articles on many of the points above.

Software and code

Policy information about availability of computer code

Data collection	BD Biosciences, software diva number v6.1.3 (cell sorting); software 8.0.2 (FACS analysis)
Data analysis	Confocal imaging analyzed by Imaris software version number 9.7.2. Statistical analysis was performed by using Graphpad Prism version number 7.0a. Nikon NIS-Elements Version 5.02.00. FIJI (ImageJ software).

For manuscripts utilizing custom algorithms or software that are central to the research but not yet described in published literature, software must be made available to editors and reviewers. We strongly encourage code deposition in a community repository (e.g. GitHub). See the Nature Research guidelines for submitting code & software for further information.

Data

Policy information about availability of data

All manuscripts must include a data availability statement. This statement should provide the following information, where applicable:

- Accession codes, unique identifiers, or web links for publicly available datasets
- A list of figures that have associated raw data
- A description of any restrictions on data availability

All raw data will be available on the YARETA server, governed by Swiss Universities. ZFIN repository ZFIN ID: ZDB-GENE-030131-8447

Field-specific reporting

Life sciences study design

All studies must disclose on these points even when the disclosure is negative.

Sample size	No statistical methods were used to determine the sample size. The sample size follows common standards (n =3 biological replicates) and similar publications in the field. Size is reported in legends for main and Supplementary figures.
Data exclusions	we excluded no data
Replication	all experiments were repeated 3 to 4 times independently. All attempts at replication were successful.
Randomization	Method of randomization was not used. Embryos by transgenic reporter lines were collected randomly only with the presence of the fluorescent proteins GFP or mcherry or the absence of both.
Blinding	There was no blinding. Because for each experiment and in particular for drug administration (H2O2, heptanol, carbenexolone, GSH, NAC, GSSG, BSO, Menadione), we (the authors) know which treatment was given to each embryo.

Reporting for specific materials, systems and methods

We require information from authors about some types of materials, experimental systems and methods used in many studies. Here, indicate whether each material, system or method listed is relevant to your study. If you are not sure if a list item applies to your research, read the appropriate section before selecting a response.

Materials & experimental systems

n/a	Involved in the study	
☐	☒	Antibodies
☒	☐	Eukaryotic cell lines
☒	☐	Palaeontology and archaeology
☐	☒	Animals and other organisms
☐	☒	Human research participants
☒	☐	Clinical data
☒	☐	Dual use research of concern

Methods

n/a	Involved in the study	
☒	☐	ChIP-seq
☐	☒	Flow cytometry
☒	☐	MRI-based neuroimaging

Antibodies

Antibodies used	Chicken anti-GFP (1:400; Life Technologies) and rabbit anti-phospho-histone 3 (pH3) antibodies (1:250,Abcam). AlexaFluor488-conjugated anti-chicken secondary antibody (1:1000; Life Technologies) and AlexaFluor594-conjugated anti-rabbit secondary antibody (1:1000; Life Technologies). Monoclonal rabbit anti-CD117/KIT antibody (Clone YR 145, Cell Marque 117R-16, dilution 1:500). Polyclonal rabbit anti-GILT/IFI30 antibody (PA5-21533, Thermo Fisher, dilution 1:5000). Monoclonal mouse anti-CD68 antibody (Clone PG M1, M0876, dilution 1:100)
Validation	All antibodies are commercially available and they have been tested by respective company. In detail: chicken anti-GFP has been validated by Life Technologies by Immunoprecipitation, immunohistochemistry (IHC) and western blot (WB) detection in multiple species (see website). Rabbit anti-phospho-histone 3 (pH3) has been validated by Abcam, by Immunoprecipitation, immunohistochemistry (IHC) and western blot, and detected in multiple species (see website). Polyclonal rabbit anti-GILT/IFI30 antibody (PA5-21533) has been validated by Thermo Fisher by Western blot analysis of GILT in human Raji lysate, and immunohistochemical analysis of GILT in the cytosol of paraffin-embedded AGS xenograft (see website). Monoclonal mouse anti-CD68 antibody (Clone PG M1, M0876) has been validated by OriGene Technologies' by formalin-Fixed, Paraffin-Embedded Human tonsil stained with CD68 using peroxidase-conjugate and AEC chromogen (see website). Monoclonal rabbit anti-CD117/KIT antibody (Clone YR 145, Cell Marque 117R-16 has been validated by Cell Marque, on human sections of formalin-fixed, paraffin-embedded tissue sections using IHC test methods (see website).AlexaFluor488-conjugated anti-chicken secondary antibody and AlexaFluor594-conjugated anti-rabbit secondary antibody have been validated by Life Technologies (see website).

Animals and other organisms

Policy information about studies involving animals; ARRIVE guidelines recommended for reporting animal research

Laboratory animals	Danio rerio, AB wild-type, ifi30-mutant, cx41.8 mutant.
Wild animals	The present study did not involve wild animals
Field-collected samples	The present study did not involve samples collected from the field.
Ethics oversight	we used only zebrafish aged less than 5 days and therefore no requirement for animal experimentation authorization

Note that full information on the approval of the study protocol must also be provided in the manuscript.

Human research participants

Policy information about studies involving human research participants

Population characteristics	Clinical case ID – Gestational age - Gender<br><br>H18008773 7 SA gender unknown - embryo<br>H14004164 9 SA F<br>H16004613 10 5/7 F<br>H14015083 11 SA M<br>H14013336 12 SA M<br>H16003204 12 5/7 M<br>H16002689 13 SA M<br>H15000848 14 4/7 M<br>H16002629 15 SA M<br>H16011109 16 2/7 M<br>H14009221 17 4/7 M<br>H14005547 18 4/7 F<br>H15013384 19 5/7 F<br>A13000202 20 2/7 F<br>A17000026 23 5/7 M<br><br>For the experiments have been used one embryo for each stage of development. Immunostainings was performed on paraffin sections.
Recruitment	Parents gave their agreement that their embryos would be used for teaching and research purposes. Fetuses were recruited according to the following criteria:<br>- Parental written approval to the use of samples for research purposes<br>- Gestational age<br>- Absence of liver pathology upon histological evaluation<br>- Adequate morphology, and in particular no tissue autolysis
Ethics oversight	CCER Commission Cantonale d'Ethique de la Recherche (Geneva)

Note that full information on the approval of the study protocol must also be provided in the manuscript.

Flow Cytometry

Plots

Confirm that:

- [x] The axis labels state the marker and fluorochrome used (e.g. CD4-FITC).
- [x] The axis scales are clearly visible. Include numbers along axes only for bottom left plot of group (a 'group' is an analysis of identical markers).
- [x] All plots are contour plots with outliers or pseudocolor plots.
- [x] A numerical value for number of cells or percentage (with statistics) is provided.

Methodology

Sample preparation	zebrafish embryos were dissociated with liberase to obtain cell suspensions
Instrument	LSR2Fortessa (analysis) and FACS AriaII (Cell sorting) and Biorad S3 (Cell sorting)
Software	DIVA to collect data, and FlowJo to analyze
Cell population abundance	purity achieved after sort was around 95% for all samples
Gating strategy	FSC/SSC gate, then exclusion of dead cells, then exclusion of doublets, before we could gate our samples of interest.

- [x] Tick this box to confirm that a figure exemplifying the gating strategy is provided in the Supplementary Information.
