# Supplementary material for: A connexin/ifi30 pathway bridges HSCs with their niche to dampen oxidative stress
Source: Nat Commun. 2021 Jul 23;12:4484. doi: 10.1038/s41467-021-24831-0 (PMC8302694; doi:10.1038/s41467-021-24831-0)
Supplement: Supplementary file 4 — Description of Additional Supplementary Files [file 41467_2021_24831_MOESM4_ESM.pdf]

Description of Additional Supplemenrtary Files

Supplementary Movie 1
Time-lapse imaging of the CHT of an *kdrl:mcherry;cmyb:GFP* embryo injected with control-morpholino (12ng, 42-48hpf).

Supplementary Movie 2
Time-lapse imaging of the CHT of an *kdrl:mcherry;cmyb:GFP* embryo injected with *ifi30*-morpholino (12ng, 42-48hpf).

Supplementary Movie 3
Time-lapse imaging of the circulation in a non-treated *globin:GFP* embryo (48hpf). Representative video from 9 embryos.

Supplementary Movie 4
Time-lapse imaging of the circulation in a heptanol-treated (50µM) *globin:GFP* embryo (48hpf). Blood circulation is comparable to the control. Representative video from 9 embryos.

Supplementary Movie 5
Time-lapse imaging of the heart in a non-treated *myl7:DsRed* embryo (48hpf). Blood circulation is slowed down compared to control and heptanol (50µM) treated embryos. Representative video from 9 embryos.

Supplementary Movie 6
Time-lapse imaging of the heart in a heptanol-treated (50µM) *myl7:DsRed* embryo (48hpf). Representative video from 9 embryos.

Supplementary Movie 7
Time-lapse imaging of the circulation in a heptanol-treated (1mM) *globin:GFP* embryo (48hpf). Heart beatings are comparable to control embryos. Representative video from 9 embryos.

Supplementary Movie 8
Time-lapse imaging of the heart in a heptanol-treated (1mM) *myl7:DsRed* embryo (48hpf). The video shows defect in heart beatings, compared to control and heptanol (50µM) treated embryos. Representative video from 9 embryos.

Supplementary Movie 9
Time-lapse imaging of the CHT in a non-treated *kdrl:mcherry;cmyb:GFP* embryo (41-48hpf).

Supplementary Movie 10
Time-lapse imaging of the CHT in a heptanol-treated (50µM, 41-48hpf) *kdrl:mcherry;cmyb:GFP* embryo.
